# Supplementary material for: Multistep nucleation and growth mechanisms of organic crystals from amorphous solid states
Source: Nat Commun. 2019 Aug 27;10:3872. doi: 10.1038/s41467-019-11887-2 (PMC6711996; doi:10.1038/s41467-019-11887-2)
Supplement: Supplementary file 1 — Supplementary Information [file 41467_2019_11887_MOESM1_ESM.pdf]

Supplementary Information for

Multistep nucleation and growth mechanisms of organic crystals from amorphous solid states

Chen *et al.*

Supplementary Methods

General Methods. All reagents and chemicals were obtained from commercial sources and used without further purification unless otherwise noted. The synthetic route is outlined in Scheme S1 and S2. All reactions were performed under an inert atmosphere of argon in dry solvents by using standard Schlenk techniques. ^1H and ^{13}C NMR spectra were recorded on Bruker–400 MHz NMR ARX400. Chemical shifts of ^1H and ^{13}C NMR signals were quoted to tetramethylsilane (δ = 0.00 ppm) and $CDCl_3$ (δ = 77.00 ppm) as internal standards, respectively. Mass spectra (MS) were recorded on a Bruker APEX IV mass spectrometer or MALDI–TOF/TOF Mass Spectrometer 5800. Elemental analyses were carried out by Elementar Vario EL CUBE (Germany).

UV–Vis absorption. UV–Vis absorption measurements of organic semiconductors in solution and in thin films (on quartz substrates) were determined with a Perkin–Elmer Lambda 950 UV/Vis spectrometer.

Cyclic voltammetry (CV). CV measurements were performed by using an electrochemical analyser (EG&G Potentiostat/Galvanostat5 model 283) in acetonitrile containing 0.001M $K_3Fe(CN)_6$ and 0.1 M KCl as a supporting electrolyte. A glassy carbon electrodes was used as a working electrode and a platinum wire as a counter electrode; all potentials were recorded versus Ag/AgCl as a reference electrode. Before measurements, the solution was deoxygenated by nitrogen bubbling for about 10 minutes. The energy levels of organic semiconductors were calculated by using the ferrocence value of –4.8 eV as the standard. The scan rate was 50 mV s^{-1}.

Thermogravimetric analysis (TGA). TGA measurements were performed on TA Instruments Q600 SDT thermal analysis system under N_2 at a heating rate of 10 ºC/min.

Differential scanning calorimetry (DSC). DSC measurements were performed by using a TA Instruments Q2000 differential scanning calorimeter under N_2.

Polarised optical microscopy (POM). POM images were obtained from monolayer films on

silicon substrates by using Nikon Eclipse LV100 POL in reflection mode.

Scanning electron microscope (SEM). Mode S4800 was used with a scan voltage of 1.0 kV.

Atomic force microscopy (AFM). The morphology of thin films was investigated by a ScanAsyst model AFM (Bruker Dimension Icon with Nanoscope V controller) under ambient conditions.

X–ray photoelectron spectroscopy (XPS). XPS data were obtained with an Axis Ultra Imaging X–ray Photoelectron Spectrometer from Kratos Analytical Ltd by using 300 W AlKα radiation. The base pressure was about 3×10^{-9} mbar. The binding energies were referenced to the C1s line at 284.8 eV from adventitious carbon.

Single crystal X–ray diffraction. The laboratory single crystal X–ray diffraction (SXRD) data for samples C_nP–BTBT (n = 3, 4, 5, 6, 9, 10 and 11) were collected at 180 K on a Rigaku Oxford XtalAB instrument (Mo Kα, λ = 0.71073 Å, graphite monochromator) and the data reduction was performed by CrysAlisPro program (version 1.171.39.9f). The synchrotron SXRD for C_nP–BTBT (n = 7 and 8) samples were collected at Beamline BL17B1 of Shanghai Synchrotron Radiation Facility (SSRF) with λ = 0.6525 Å at 100 K and the data were processed with HKL3000 software. The structure was solved by direct methods and refined by minimizing the sum of squared deviations against F^2 using a full-matrix technique with the SHELXL–97 program[1].

Film X–ray diffraction. Film X–ray diffraction data were collected on PANalytical high resolution PXRD.

Grazing–incidence X–ray diffraction (GIXD). GIXD data were obtained at beamline BL14B1 of the Shanghai Synchrotron Radiation Facility (SSRF) at a wavelength of 1.2398 Å. BL14B1 is a beamline based on bending magnet and a Si (111) double crystal monochromator was employed to monochromatise the beam. The size of the focus spot is about 0.5 mm and the end station is equipped with a Huber 5021 diffractometer. A NaI scintillation detector was used for data collection. The *d*–values of diffraction spots were integrated by a FIT2D software.

Morphology calculation. According to the single crystal structures of all these molecules, crystals' morphologies for different molecules were calculated by the Morphology modules in Materials Studio v7.0. The force field of Universal was used with Use current charges. The Crystal Graph method was used to calculate growth morphology with medium quality.

High–resolution fluorescent imaging. First, quartz chips used as substrates were ultrasonised for 10 min by ethanol, acetone and deionised water, respectively, and dried by an inert gas of nitrogen. Then, we made a 1 mg/mL C_7P–BTBT solution in chloroform containing 1×10^{-4} mg/ml CP–2PhAn and ultrasonised it for 10 minutes. After that, the mixed solution was spin coated on quartz chips.

We imaged the samples by using the conventional mode of a Nikon Stochastic Optical Reconstruction Microscopy (N–STORM) with a 100 × TIRF oil immersion objective (NA 1.49) and 8% laser (λ = 405 nm) excitation power. We continuously imaged the samples for 48 h. The exposure time was 200 ms and the interval was 3 min. The imaging data were processed with ImageJ, and the video files were saved under the same contrast after the drift correction.

Device Fabrication and Characterisation. Top-contact/bottom-gate (BG/TC) microwire devices were fabricated *in–situ* n^+-Si/SiO_2 substrates where n^+-Si and SiO_2 were used as the gate electrode and gate dielectric, respectively. The gold source and drain contacts (100 nm) were glued on the substrate onto the surface of the microwire. The channel length (L) and width (W) were 30 μm and 1.5 μm, respectively. The mobilities of the devices were calculated in the saturation regime by the standard method:

$$I_{DS} = (W/2L)C_i\mu(V_G - V_T)^2$$

Where W/L is the channel width/length, and V_G and V_T are the gate voltage and threshold voltage, respectively. The transistor characteristics were obtained at room temperature in air by using a standard probe station and semiconducting parameter analyser (Agilent 4155C).

Supplementary Figures

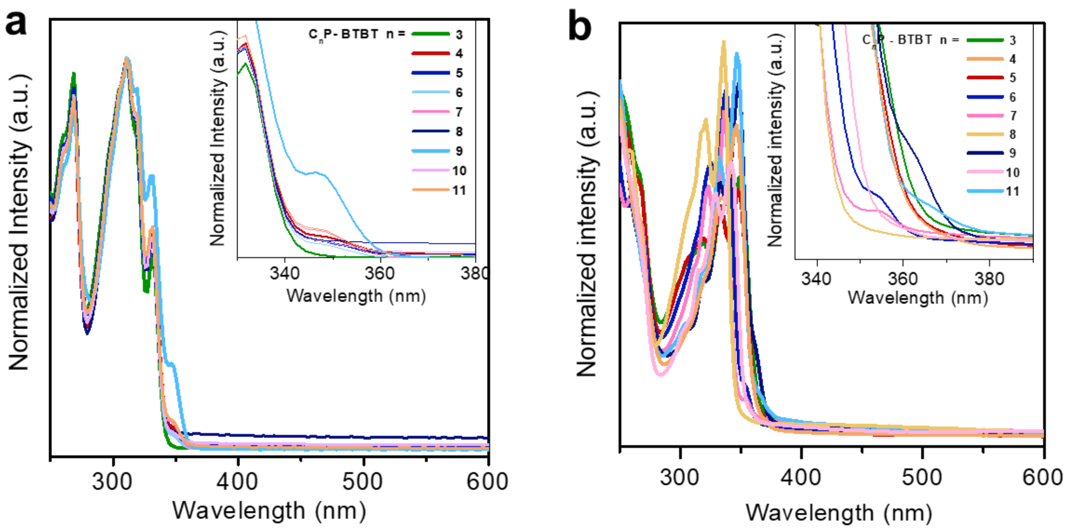

Supplementary Figure 1 | Normalised UV–Vis absorption spectras of C_nP–BTBT (n = 3 to 11). **a** UV–Vis absorption of the solutions (10^{-5} M, $CHCl_3$). **b** UV–Vis absorption of the thin fims on quartz substrates. Insets show the on-set absorption.

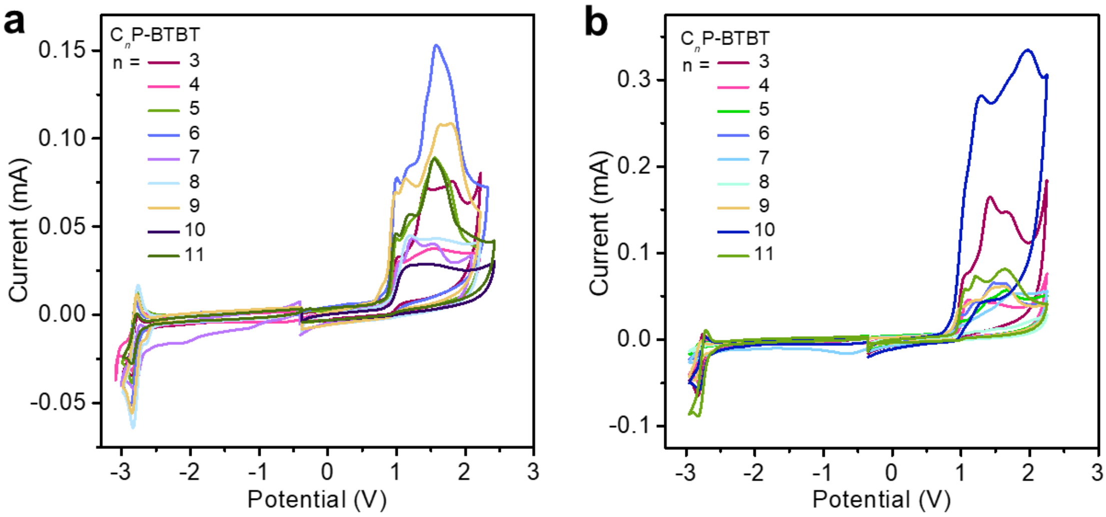

Supplementary Figure 2 | Cyclic voltammogram (CV) of C_nP-BTBT (n = 3 to 11). **a** CV spectras in CH_3CN solution. **b** CV spectras of thin-films drop-cast on a glassy carbon electrode and tested in Bu_4NClO_4/CH_3CN solution (scan rate, 50 mV s^{-1}).

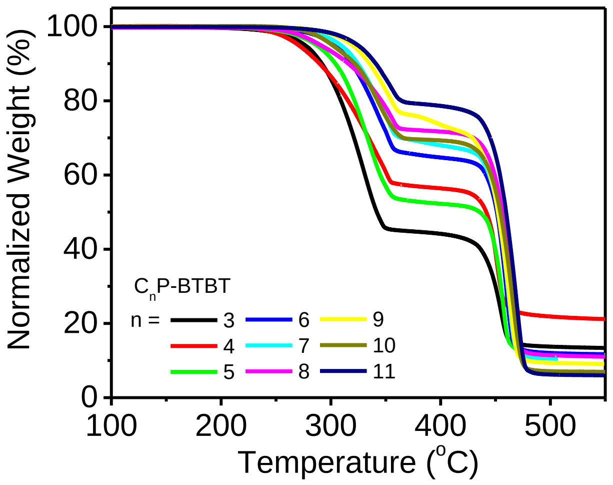

Supplementary Figure 3 | TGA curves of C_nP-BTBT (n = 3 to 11).

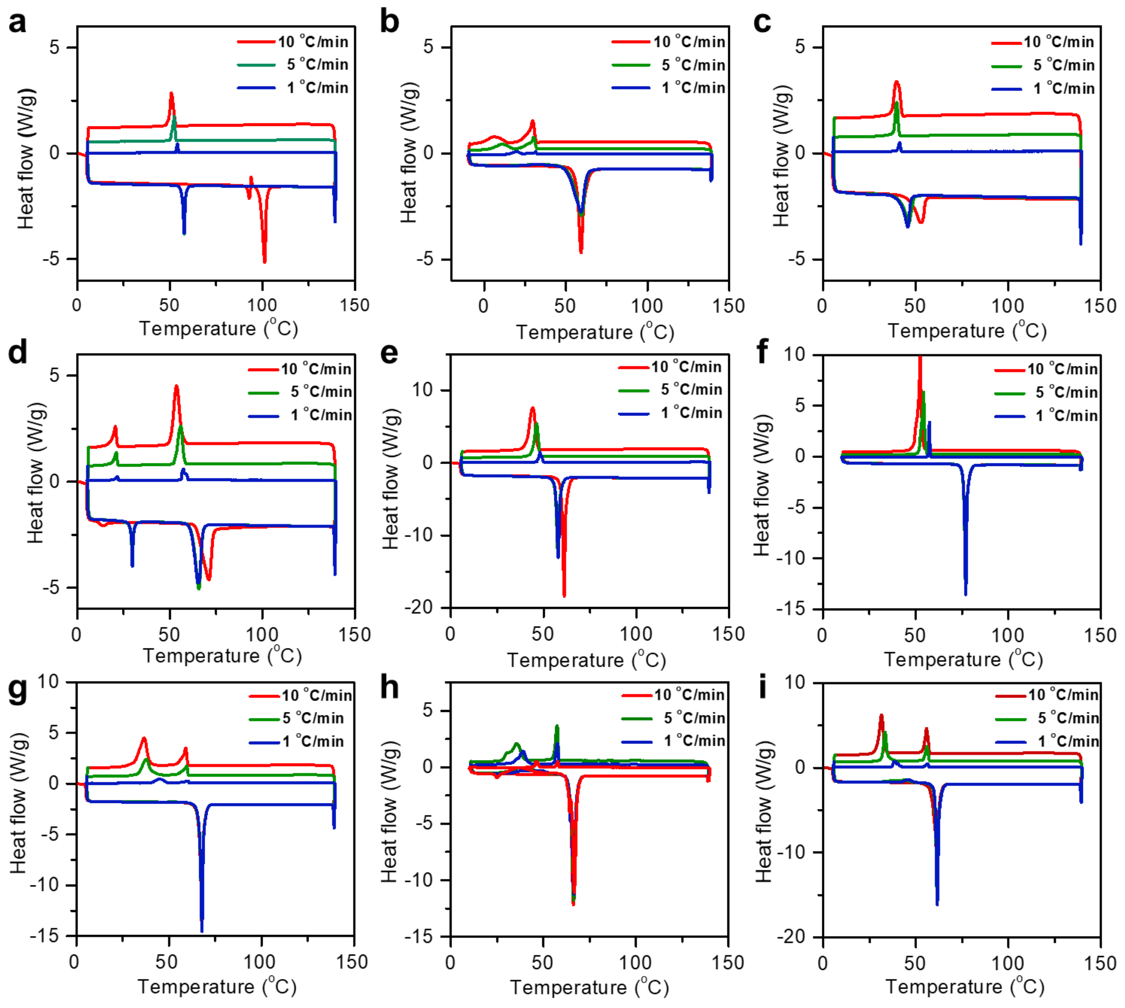

Supplementary Figure 4 | Differential scanning calorimetry (DSC) traces of C_nP-BTBT. **a** to **i** correspond to n = 3 to 11 (Scan rate: 1, 5, and 10 ºC/min).

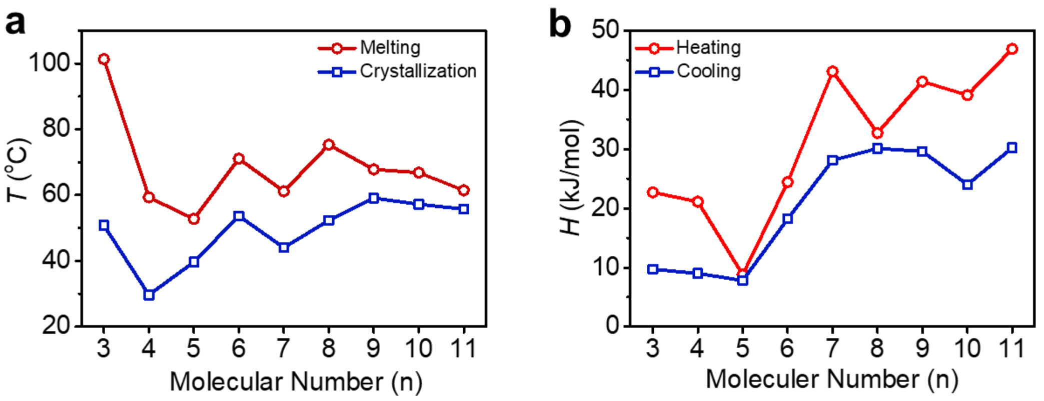

Supplementary Figure 5 │ Thermal properties of C_nP-BTBT. **a** Phase transition temperatures extracted from DSC thermograms. **b** Enthalpy changes obtained during heating and cooling cycles.

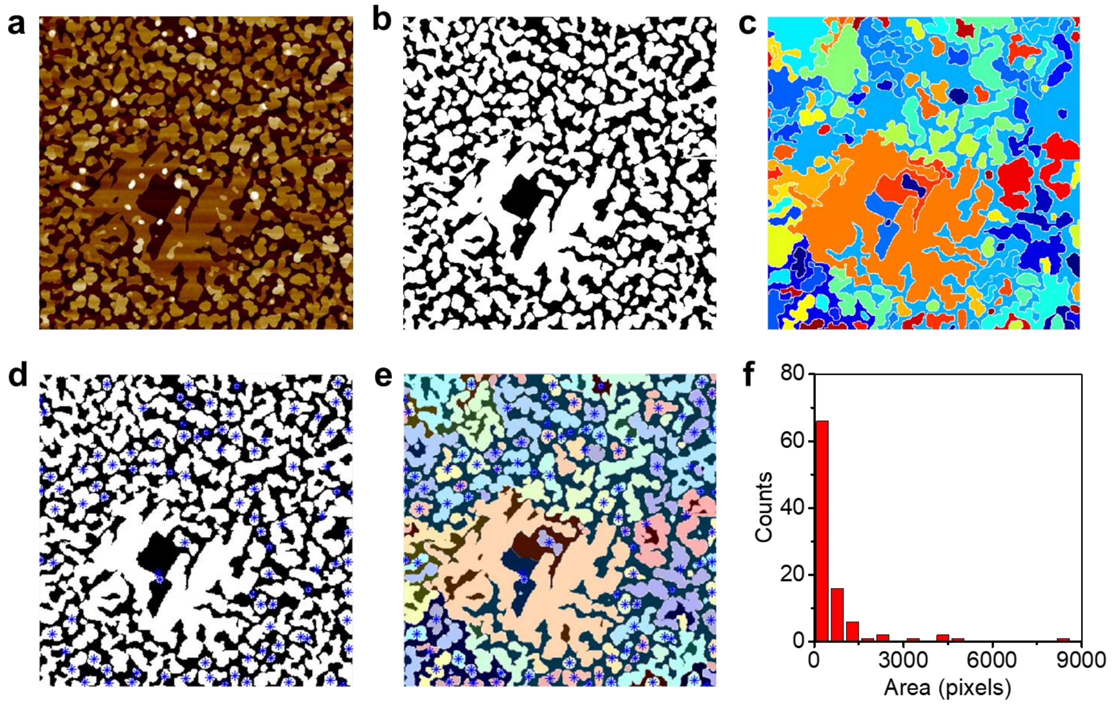

Supplementary Figure 6 │ Marker-based watershed segmentation. **a** Original AFM image. **b** Binary image. **C** Watershed transformation image. **d** Thresholding operation to find the edge. **e** Use transparency to superimpose the pseudo-color label matrix on top of the original AFM image. **f** Histogram of area counts.

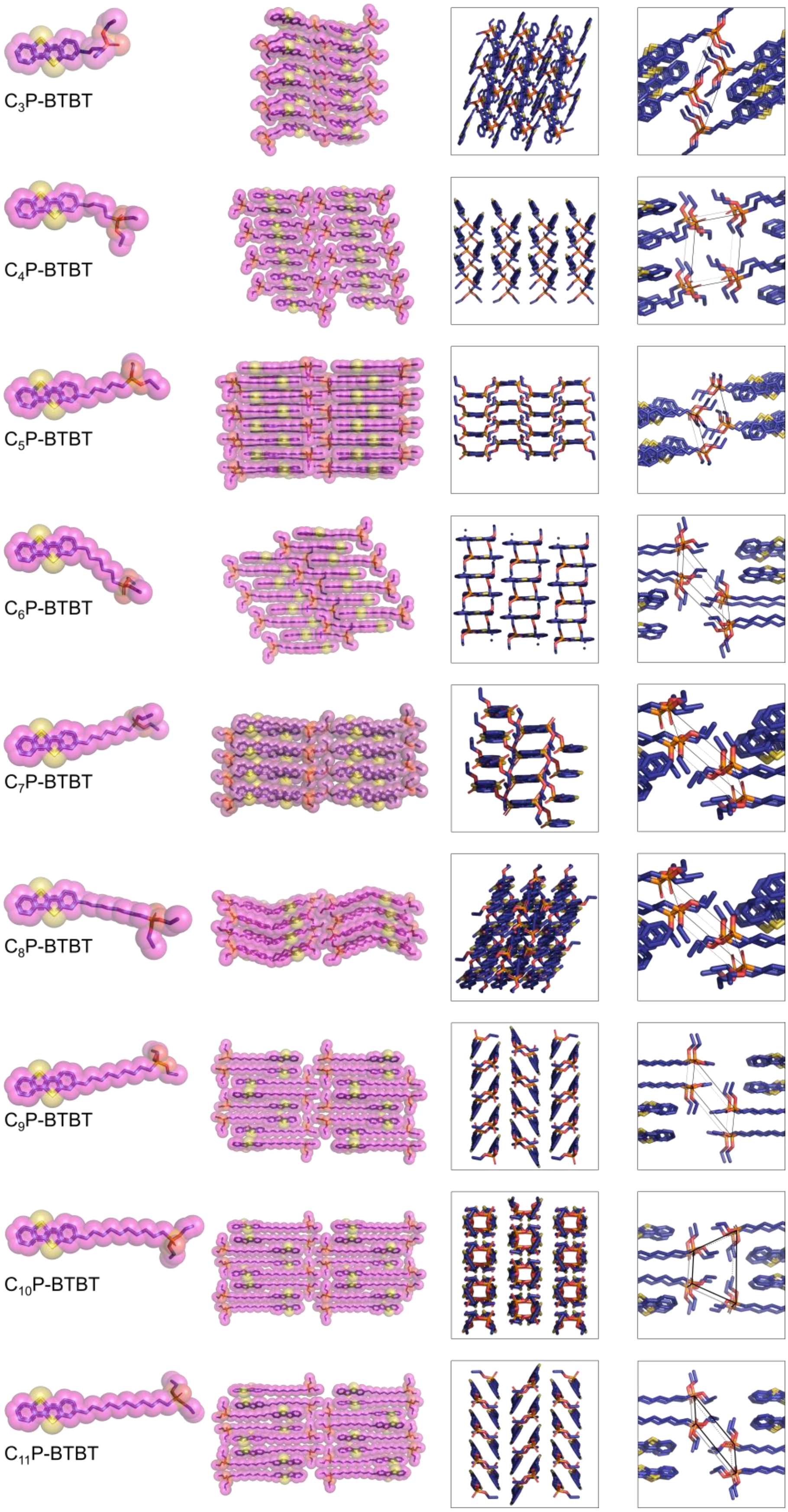

Supplementary Figure 7 | Single crystal structures of C_nP–BTBT (n = 3 to 11).

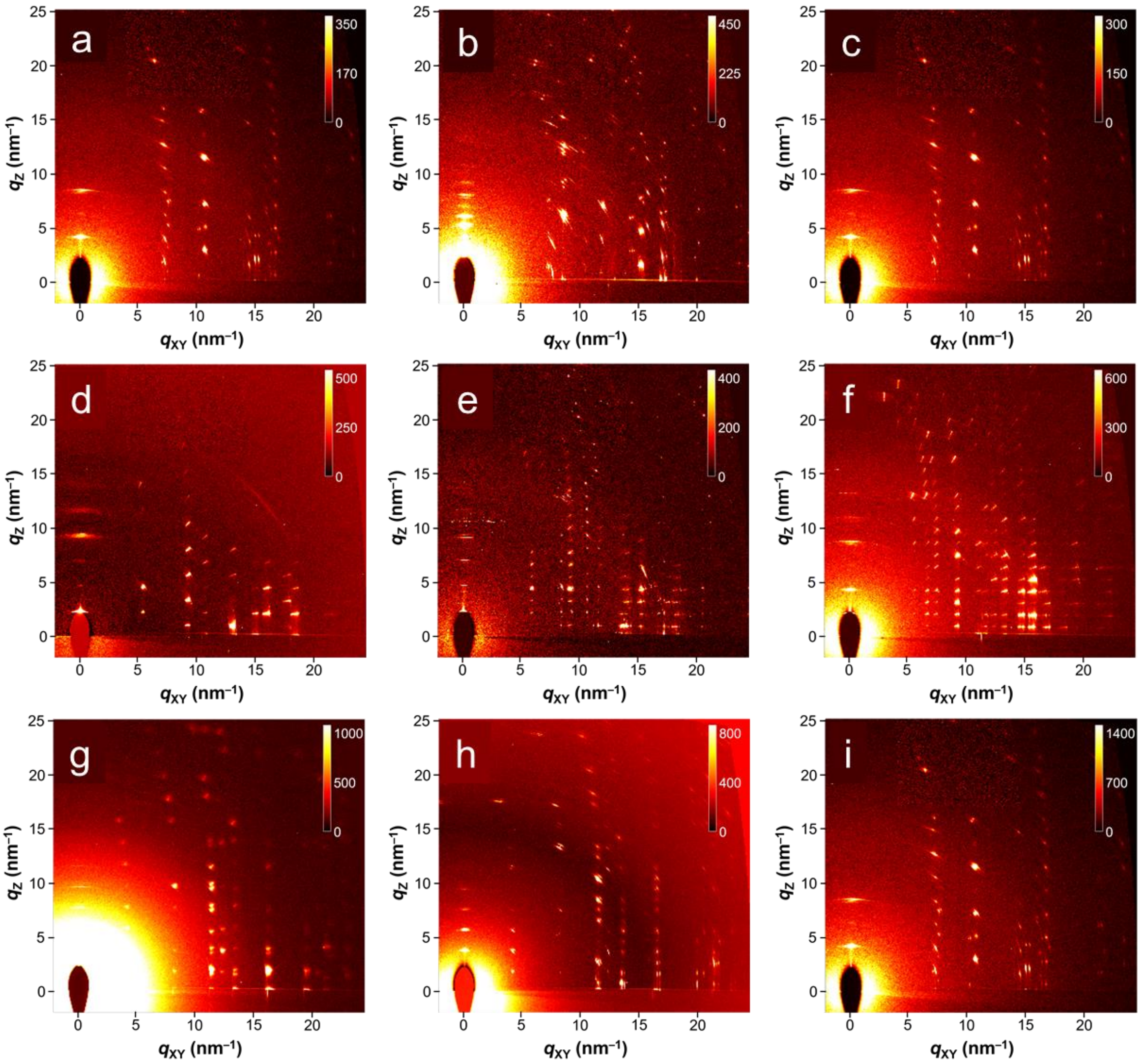

Supplementary Figure 8 │ 2D-GIXD patterns of C_nP-BTBT (n = 3 to 11). The films were kept for 48 hours before measurements. They can be regards as film-microwire hybrids. **a** to **i** correspond to n = 3 to 11.

To explore the growth orientation of these molecules, we tested the GIXD of these molecules C_nP–BTBT (n = 3 to 11) and computed the crystal morphology to find maximum exposure crystal faces. The corresponding *d*–values of every adjacent spots in GIXD patterns relative to vertical and horizontal directions could be calculated by an integration method through a FIT2D program. Then, the corresponding faces of these *d*–values could be searched by referring to crystal lattices and structures. Table S5 shows the dominant faces from computations and GIXD patterns. Through GIXD patterns, we could verify one dominant face from C_3 to C_7, and two dominant faces from C_8 to C_{11}. This means that for C_8 to C_{11}, the molecules could form more regular arrangements. The 2D-GIXD measurements also verify that C_7P-BTBT microwires stack with (100) planes in the normal direction of substrates and grow along (010) in the longitudinal direction.

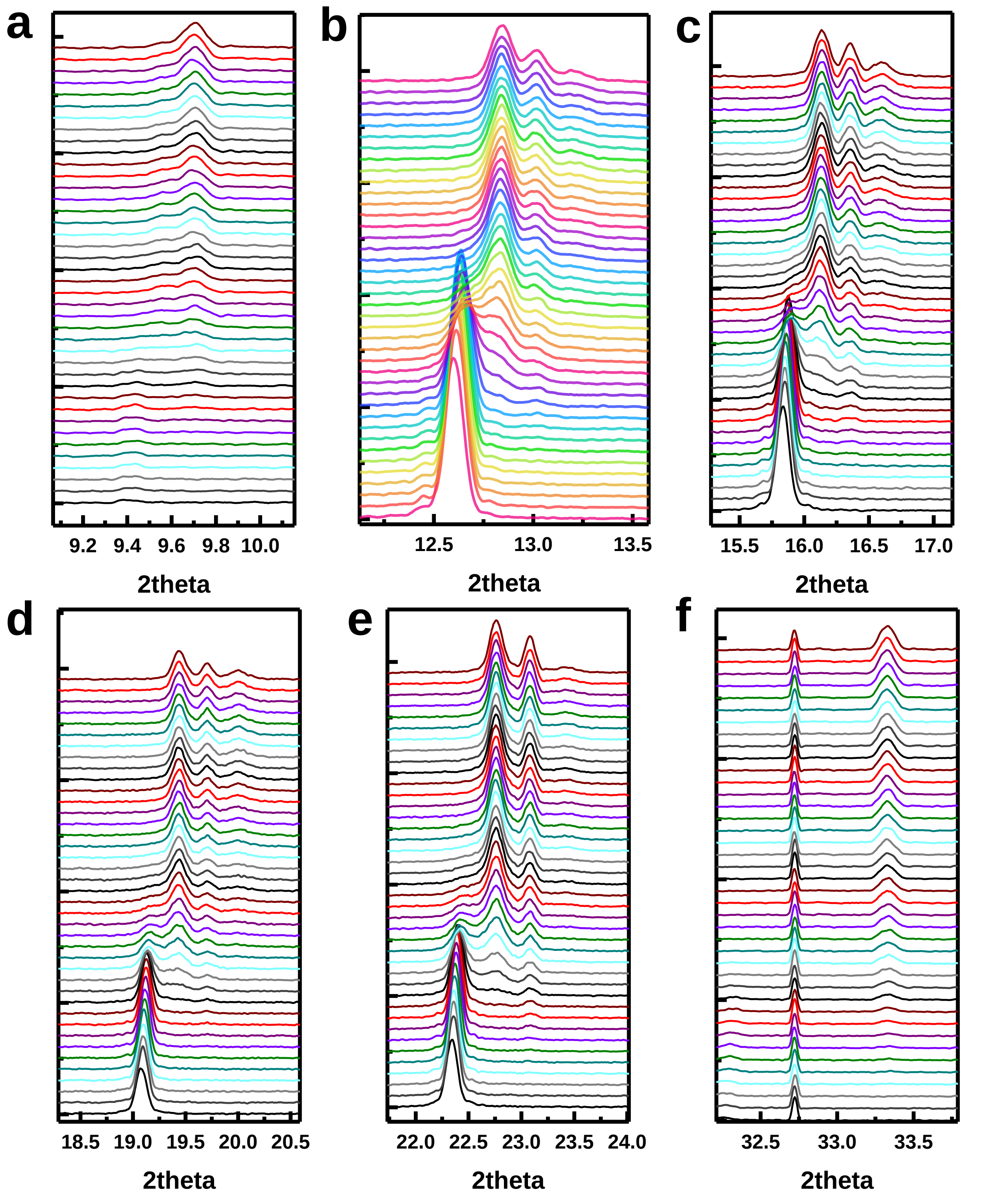

Supplementary Figure 9 │ Time-dependent film XRDs of C_7P–BTBT at different angles**. a** to **f** corrosponds to peak (040), (060), (080), (0100), and (0180), resectively.

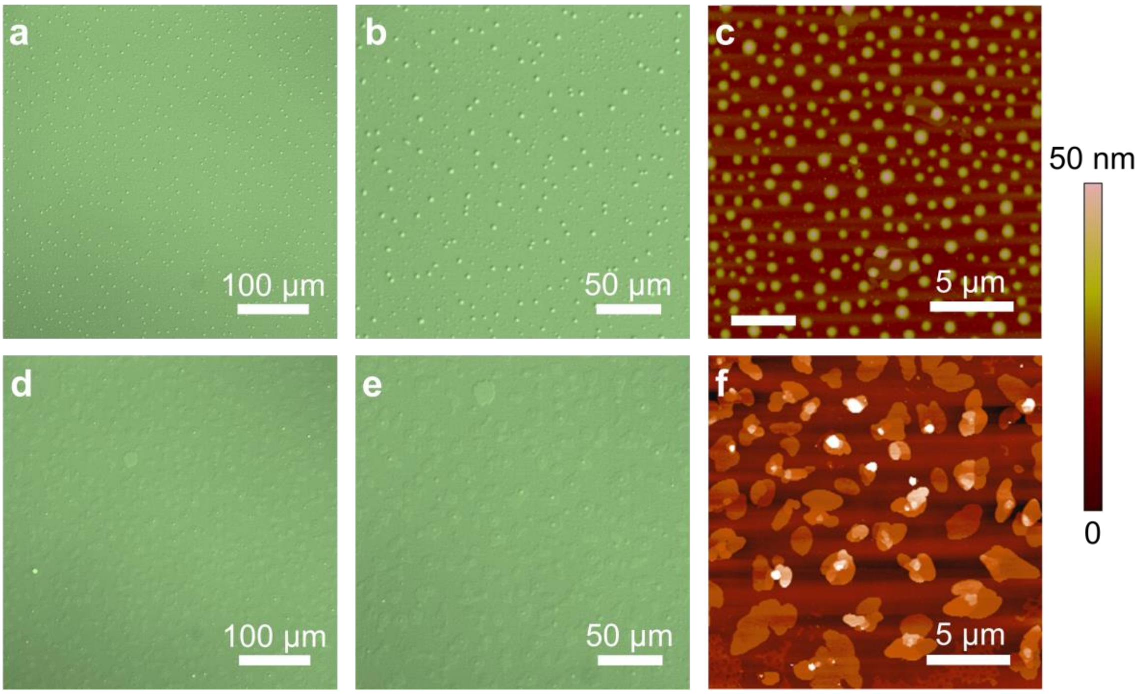

Supplementary Figure 10 | The prenucleation process. **a, b** Optical microcsopic (OM) images. **c,** AFM image of as-cast liquid–like droplets. **d, e** OM images. **f** AFM image showing the pancake–like morphology of the dwetting films.

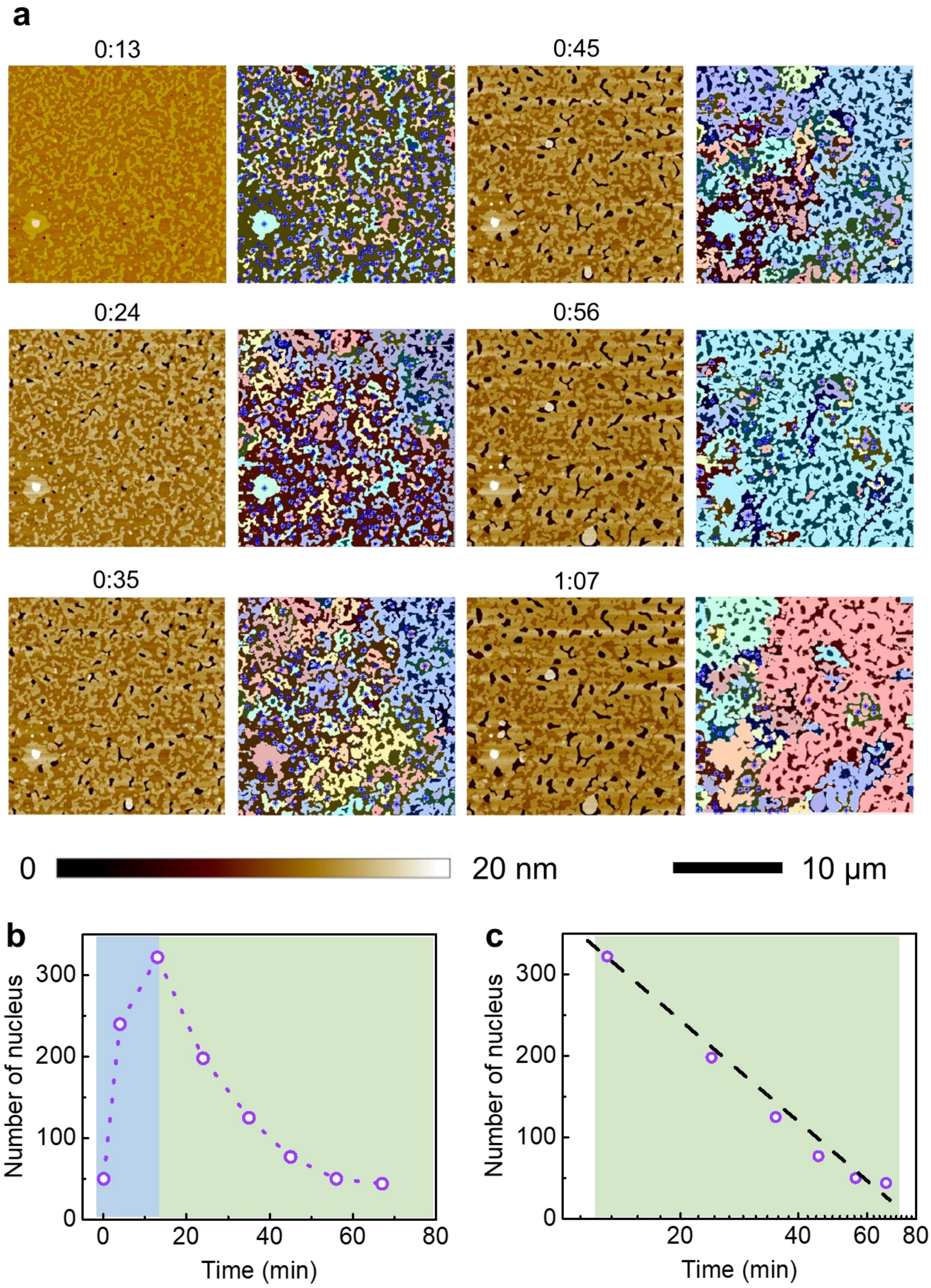

Supplementary Figure 11 | Nucleation of thick islands from base film. **a** Time-lapse sequence of AFM images (left) and watersed segmentation (right), showing the evolution of thick islands during the prenucleation process. **b, c** Linear (**b**) and Semilogarithmic (**c**) relationship of thick island numbers versus growth time for the ensemble thick islands in (**a**). The black dash line in (**c**) shows a linear fit.

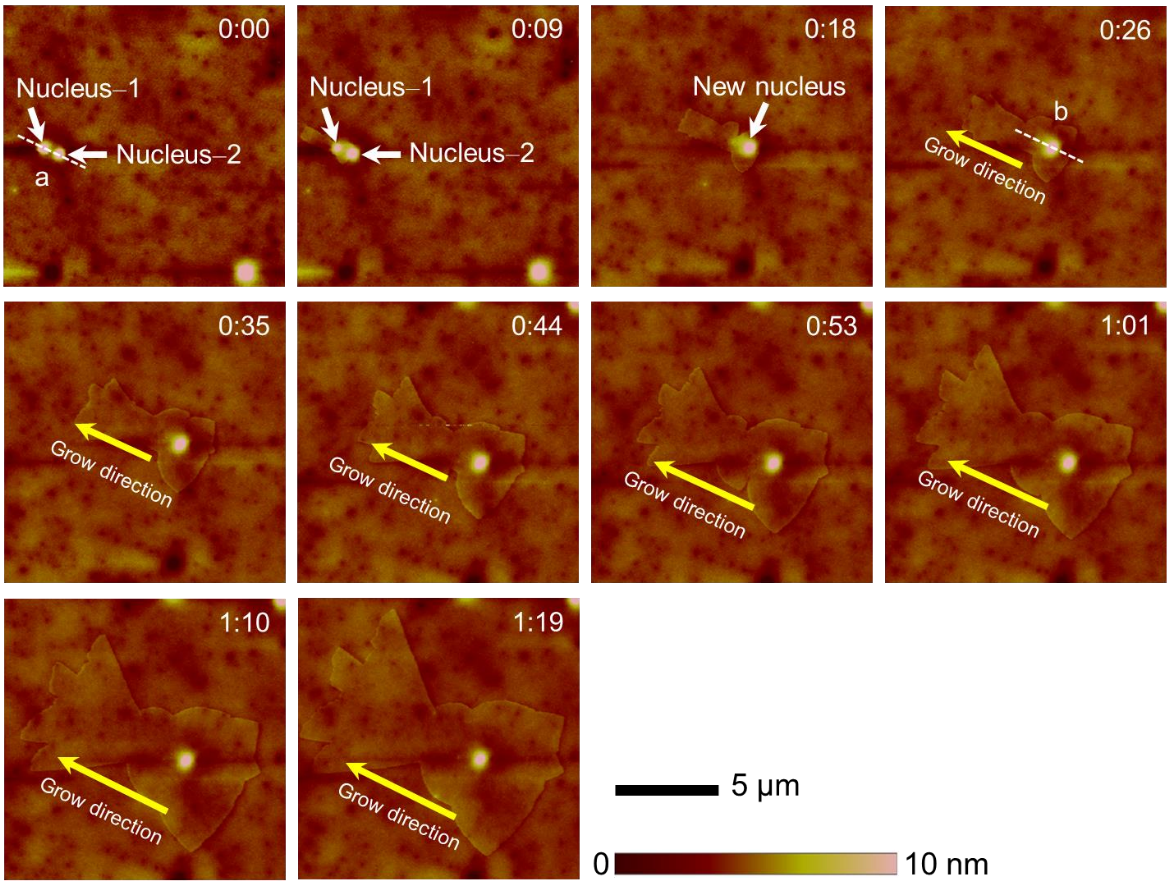

Supplementary Figure 12 │ Evolution of liquid-like droplet diameter and desolventised film area during the prenucleation process on quartz substrate. Pancake-like desolventised film also forms on quartz substrate. Coalensence of two droplets (Nucleus–1 and Nucleus–2) occurs at 0:00 and totally merges at t = 18 min (New nucleus).

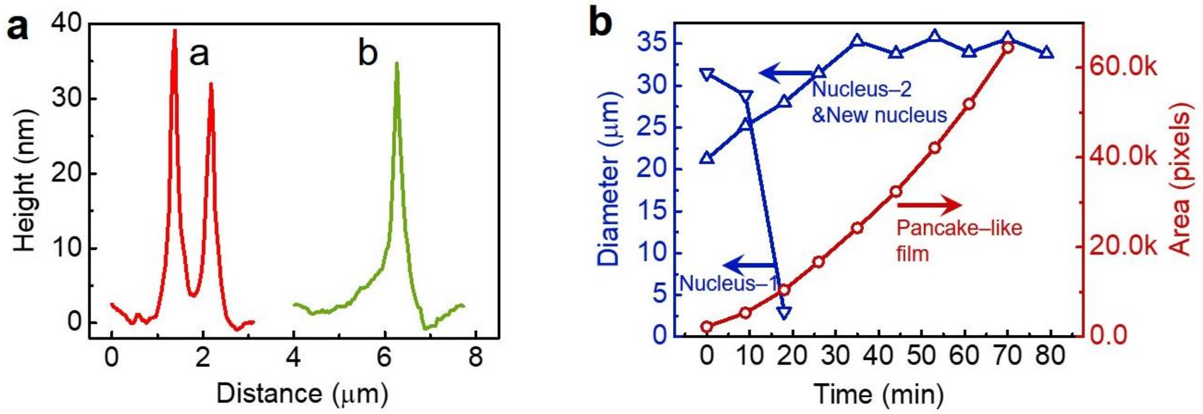

Supplementary Figure 13 │ Evolution statistics. **a** Height profile of Nucleus–1, Nucleus–2, and the new nucleus as shown in Supplementary Figure 12. **b** Dimeter evolution of nuclei and area evolution of pancake-like film versus time as highlighted in Supplementary Figure 12.

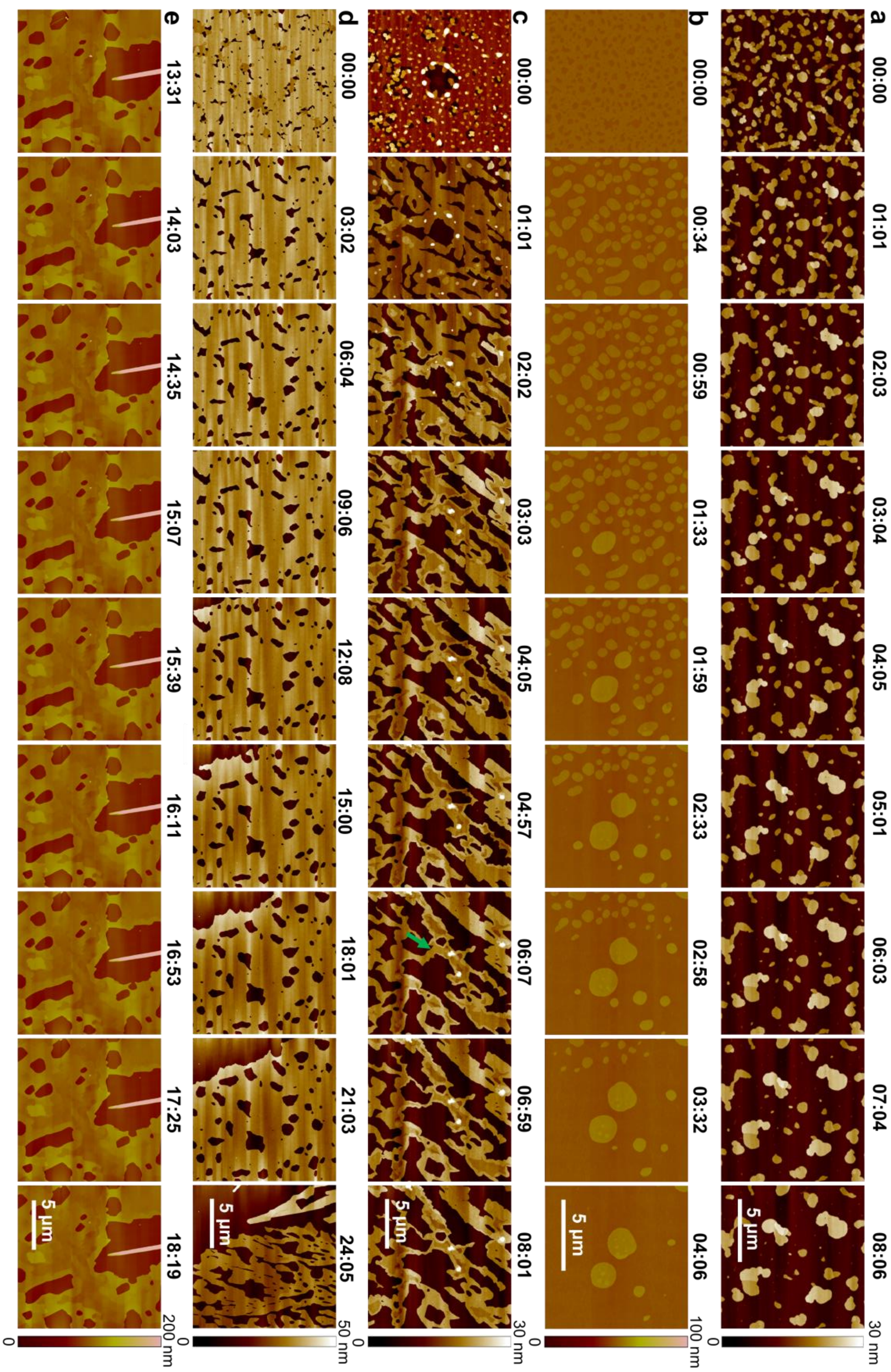

Supplementary Figure 14 | Concentration-dependent experiments. **a** 0.5 mg/mL; **b** 0.75 mg/mL; **c** 1.0 mg/mL; **d** 2.0 mg/mL; **e** 4.0 mg/mL.

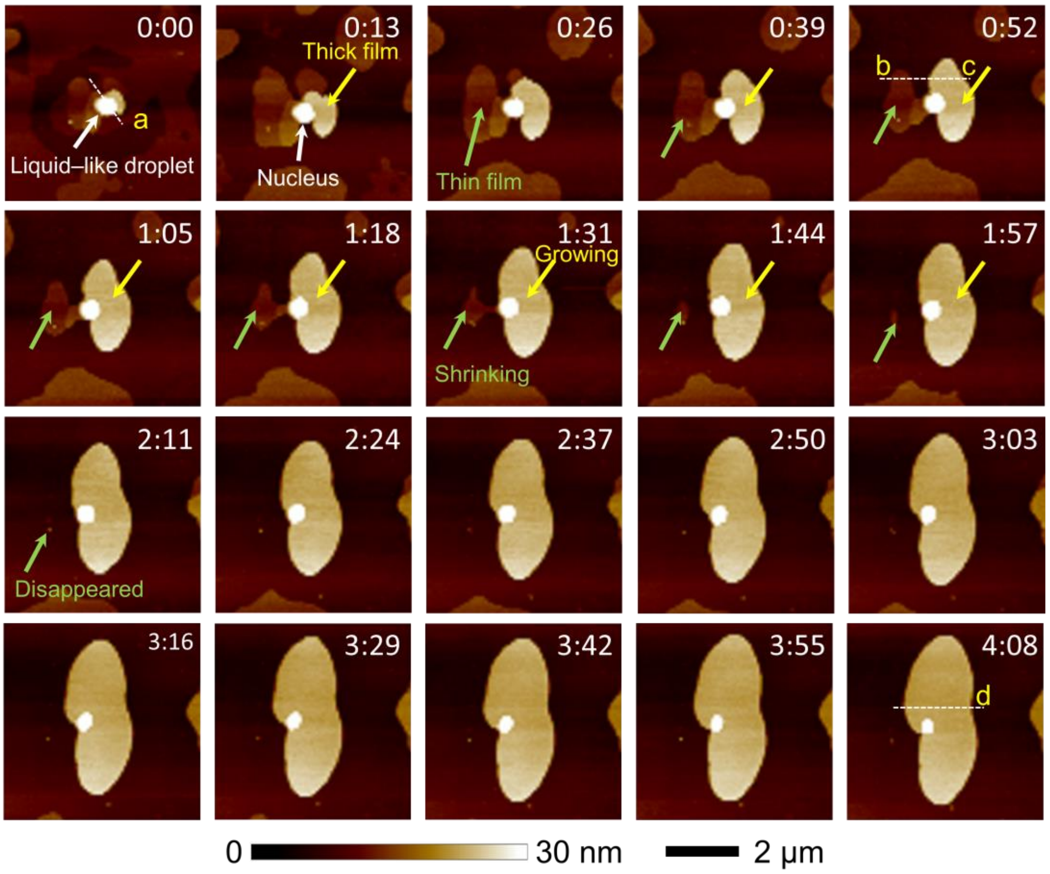

Supplementary Figure 15 | Evolution of liquid-like droplet diameter and desolventised film area during the prenucleation process. We can clearly see that the dwetting of the droplet forms the pancake-like film. After that, coalensence of two flims (thick film and thin film) occurs at t = 13 min and the thin film totally disappeares at t = 2 h 11 min.

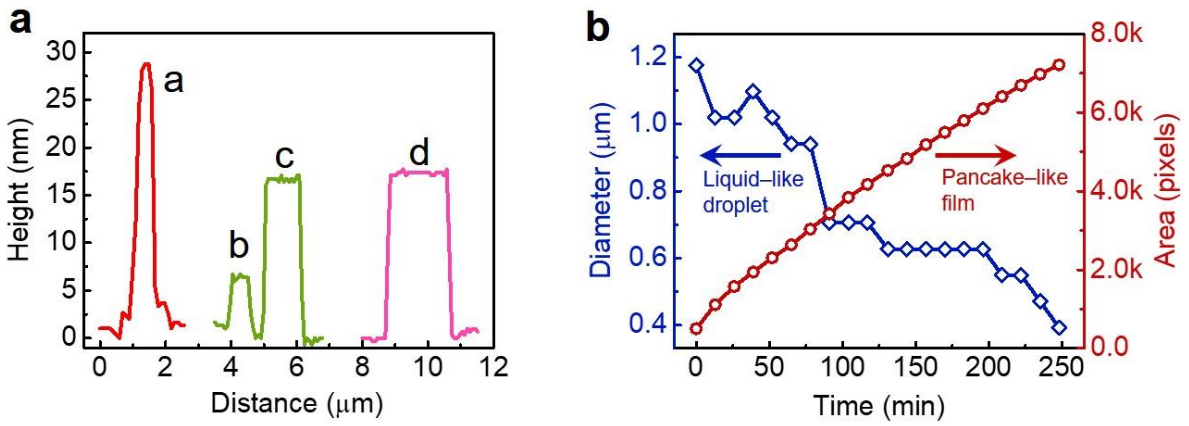

Supplementary Figure 16 | Evolution statistics. **a** Height profile cross the edge from the substrate to each layer as shown in Supplementary Figure 15. **b** Film area versus time. The films are highlighted in Supplementary Figure 15.

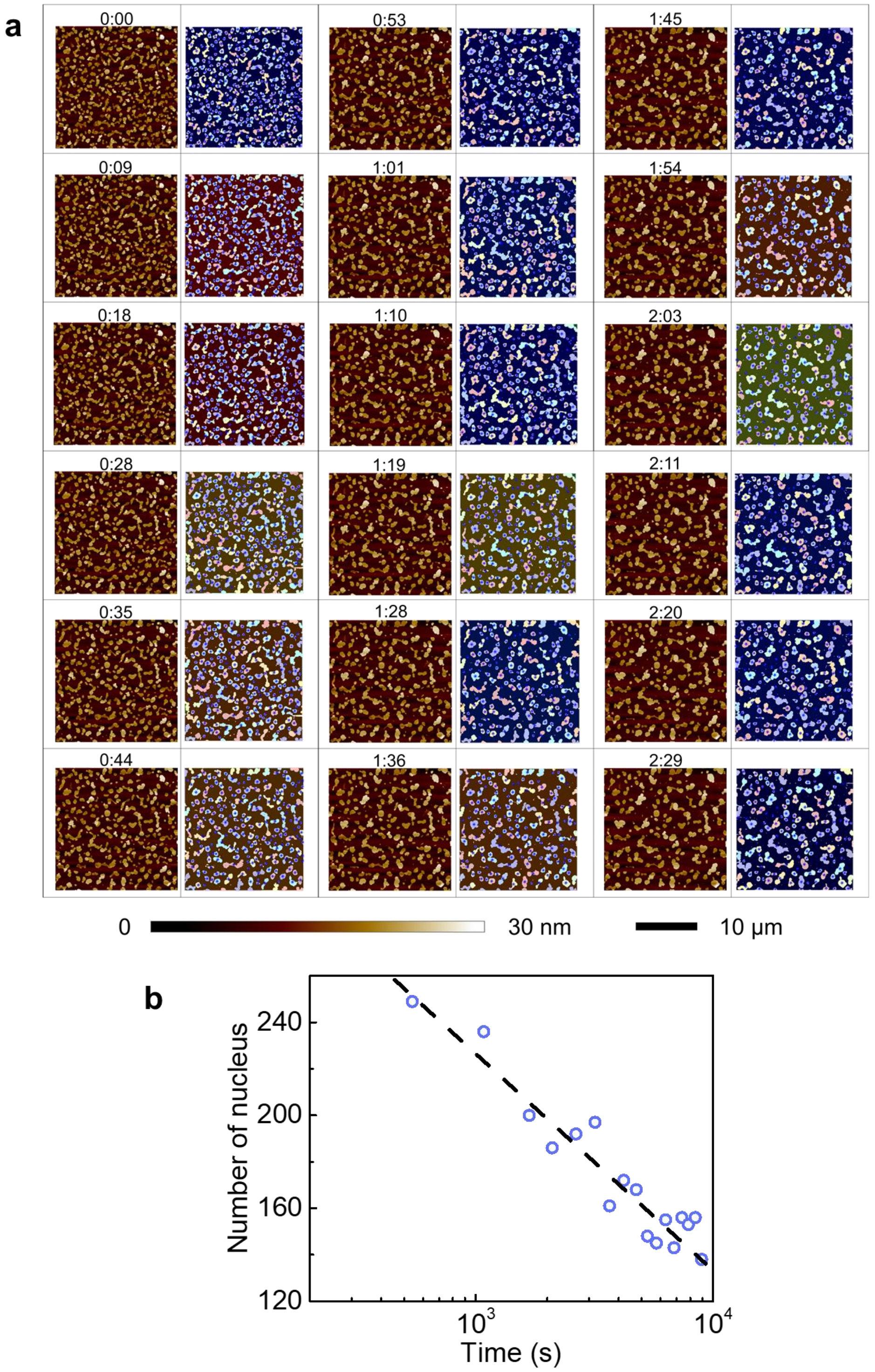

Supplementary Figure 17 │ Coallencence of desolventised nanoplates during the prenucleation process. **a** Time-lapse sequence of AFM images (left) and watersed segmentation (right) showing the coallencence events of nanoplates during the prenucleation process. **b** Semilogarithmic relationship of nanoplate numbers versus growth time for the ensemble and those individual nanoplates in (**a**). The black dash line shows a linear fit.

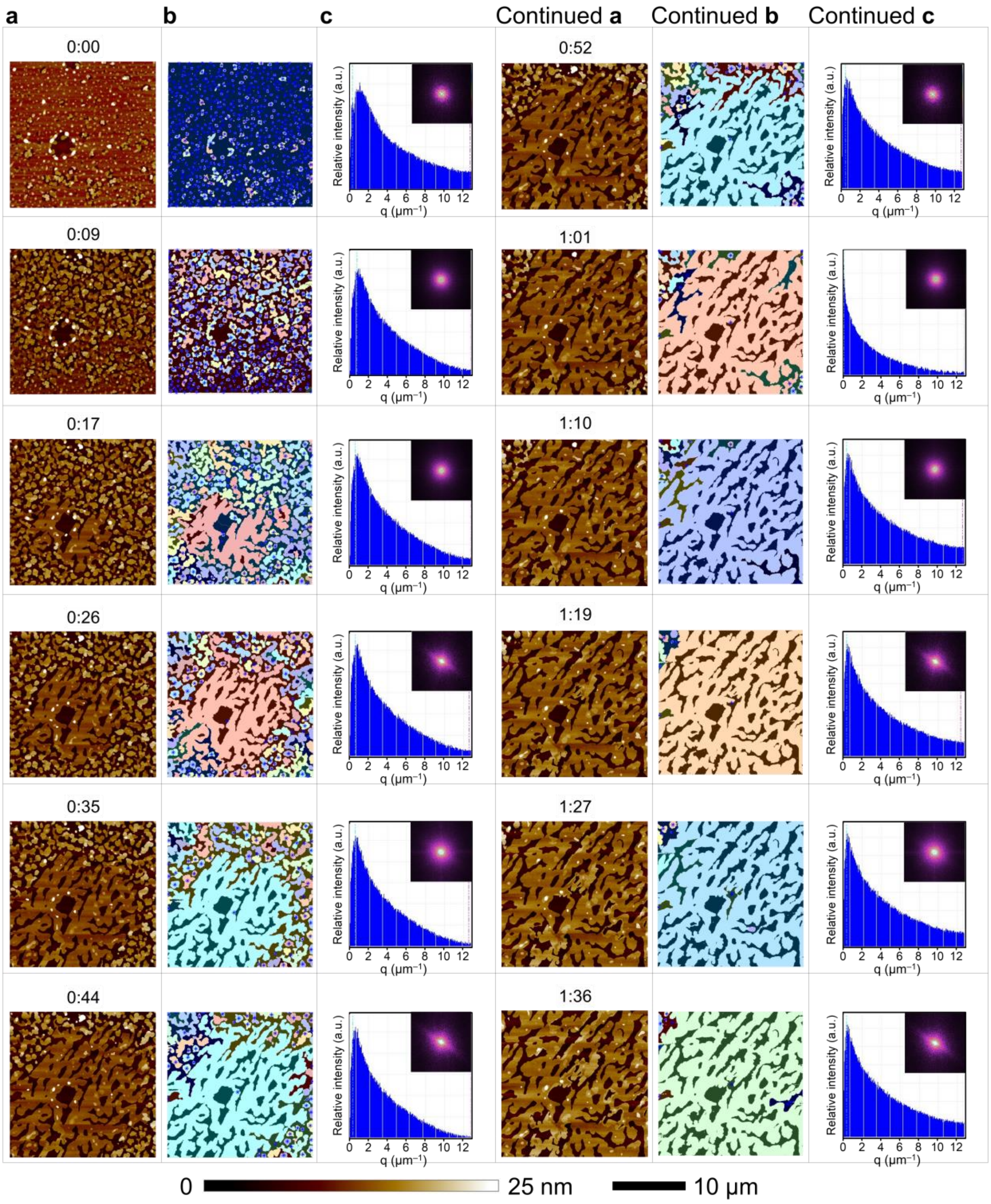

Supplementary Figure 18 │ Spinodal decomposition of fully–covered base film. **a** Time–lapse sequence of AFM images. **b** Watersed segmentation showing the spinodal decomposition events between Stage 1 and Stage 2. **c** Plots of the relative intensity versus wave vector q. Intensity plots were obtained from radial averages of 2D FFT images as shown in right insets.

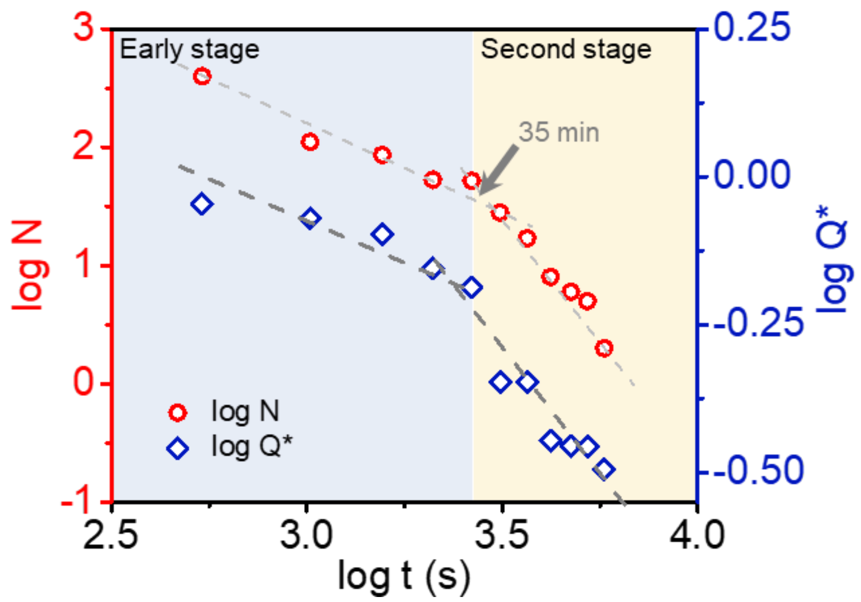

Supplementary Figure 19 | Logarithmic relationship of islands number N and Q* versus time, showing a two-stage film break-up process. A peak in the scattering intensity is observed at $q = Q^*$, determining the scale of the in–plane surface undulations. Gray dash lines show linear fits.

A capillary wave model has been built for investigating thin–film dewetting from solid substrates. This model predicts the growth of small surface undulations $Z(x, t)$:

$$Z(x, t) = h + \delta h\, exp(iqx) \quad \delta h = \delta h_0\, exp(Rt)$$

with film thickness h, fluctuation amplitude δh, growth rate R, and time t. The coordinate x is taken to be parallel to the surface and q denotes the wave vector. A peak in the scattering intensity is observed at $q = Q^*$, determining the scale of the in–plane surface undulations (Supplementary Figure 18). The relative intensity plot versus the wave vector q was obtained from the radial average of the 2D fast Fourier transform (FFT) image. A plot of $\log Q^*$ versus $\log t$ suggests two stages in the spinodal dewetting process (Supplementary Figure 19). In the first 35 min (early stage), Q^* varied slowly with time, very similar to the early stage of spinodal decomposition in blends. In the second stage, after the breakup of the films into oriented films, the surface structure coarsened rapidly and the growth could be described by an apparent power law with an exponent near –0.8. The estimated value of this exponent is notably similar to that obtained for polymer phase separation in ultrathin blend films.

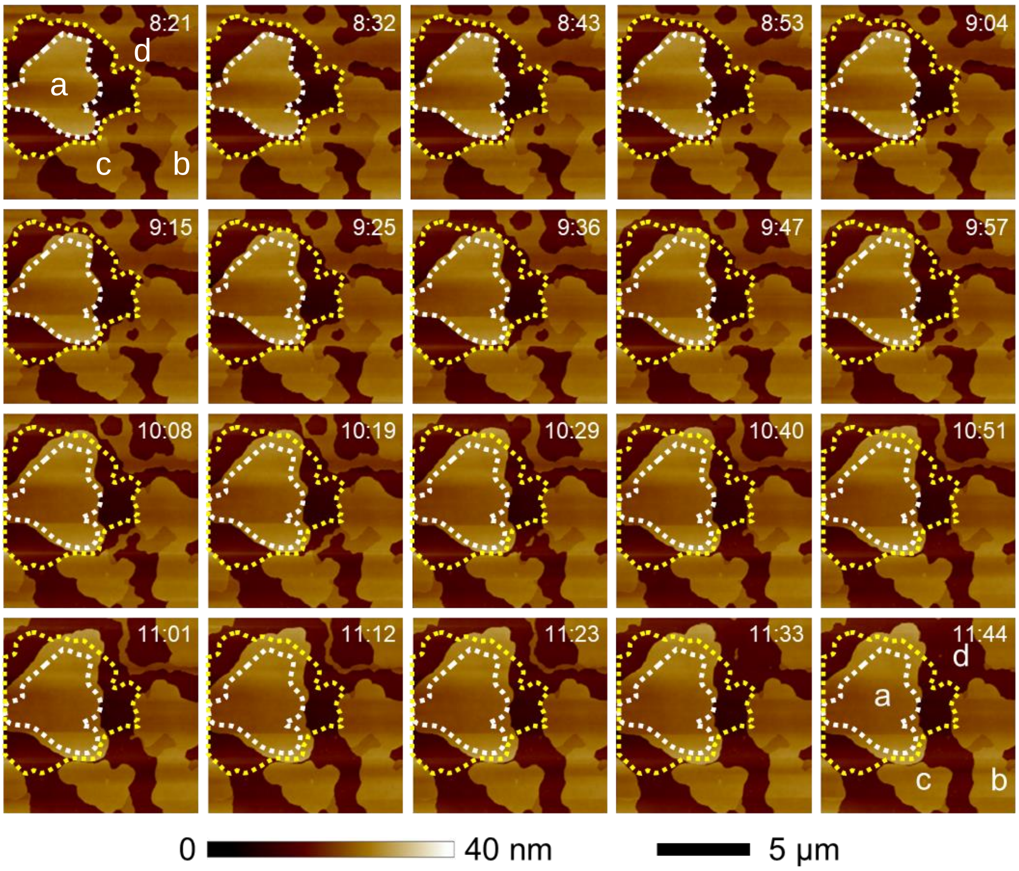

Supplementary Figure 20 | Mass transport from thin films to thick films (Sample 1). Domain a, b, and c are small–volume thick films; while Domian d is a large–volumn thin film. We can clearly see from the time-lapse sequence AFM images that Domain a, b, and c grow at the expense of Domain d. Domain d totally disappearly at t = 11 h 44 min.

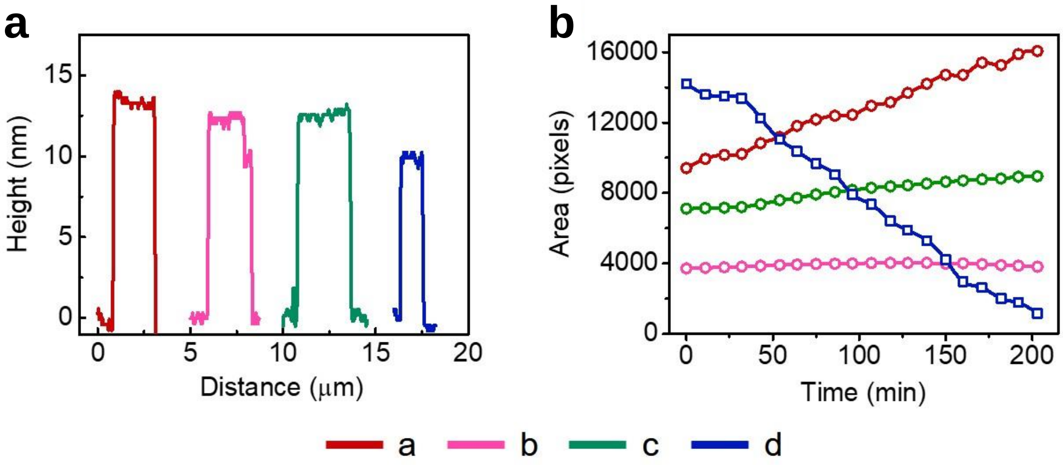

Supplementary Figure 21 | Evolution statistics. **a** Height profile cross the edge from the substrate to each layer as shown in Supplementary Figure 20 (t = 8 h 21 min). **b** Film area versus time. The films are highlighted in Supplementary Figure 20.

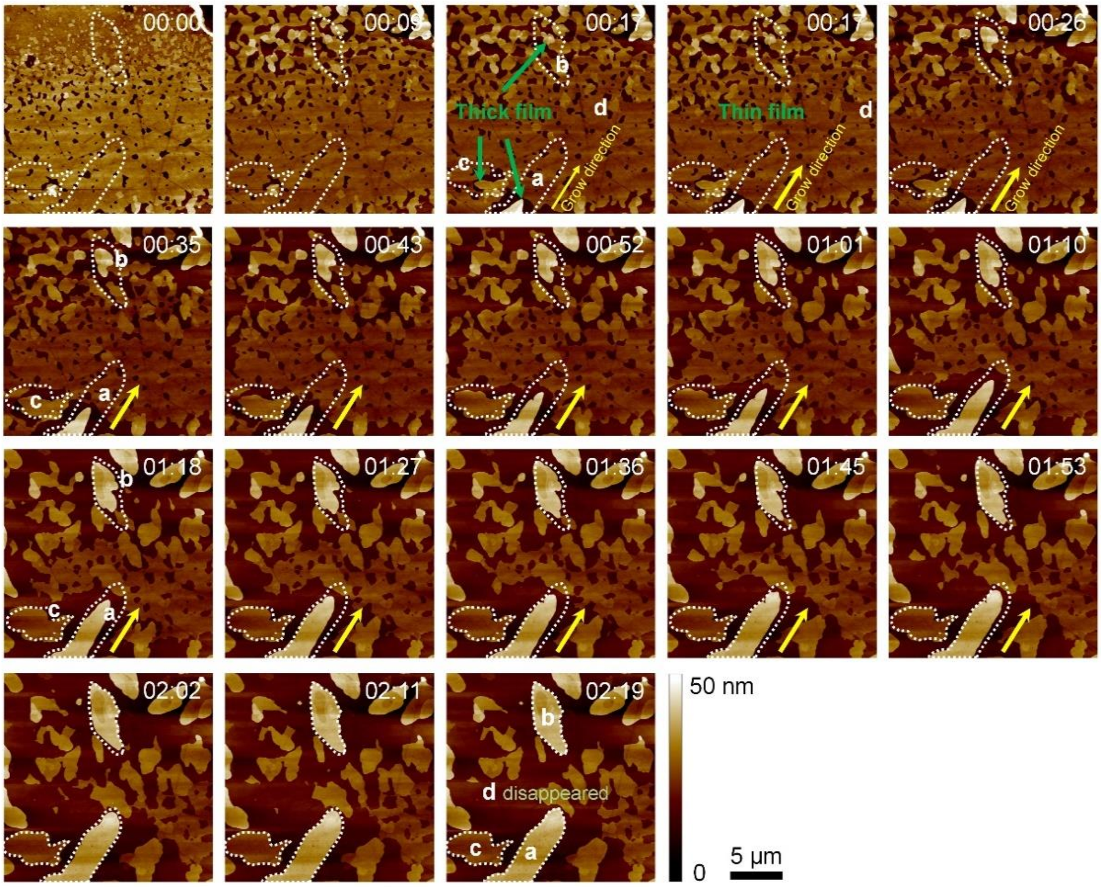

Supplementary Figure 22 │ Time-lapse sequence of AFM images showing the layer and lateral growth mode on quartz substrate (Sample 2). We can clearly see from the AFM images that thick films (Domain a, b, and c) are growing while thin film (Domian d) is shrinking into discrete films at t = 2h19min.

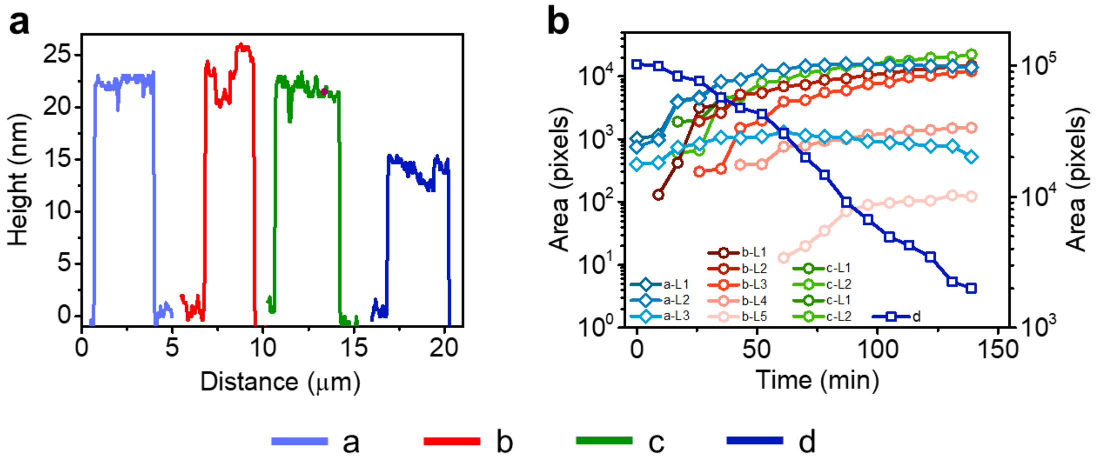

Supplementary Figure 23 │ Evolution statistics. **a** Height profile of thick films (Domain a, b, and c) and thin film (Domain d) as shown in Supplementary Figure 22. **b** Film area versus time. The films are highlighted in Supplementary Figure 22.

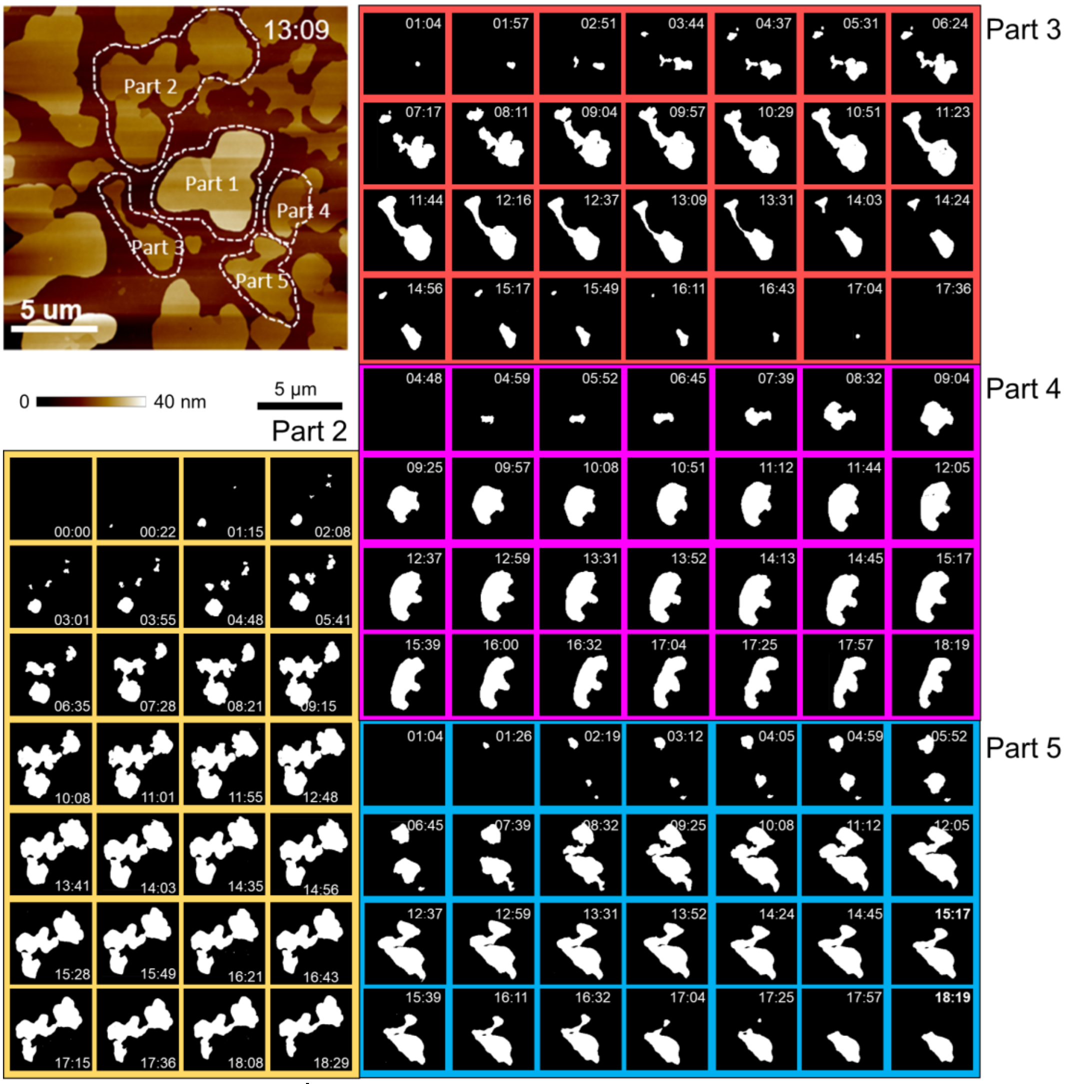

Supplementary Figure 24 │ Evoluation of thin films (Parts 2, 3, 4 and 5) showing a downward parabola relation of domain size (Sample 3). They undergo a size increase in the early stage while scarifying themselves in the late stage through Ostwald ripening to grow thick films (**Part 1**).

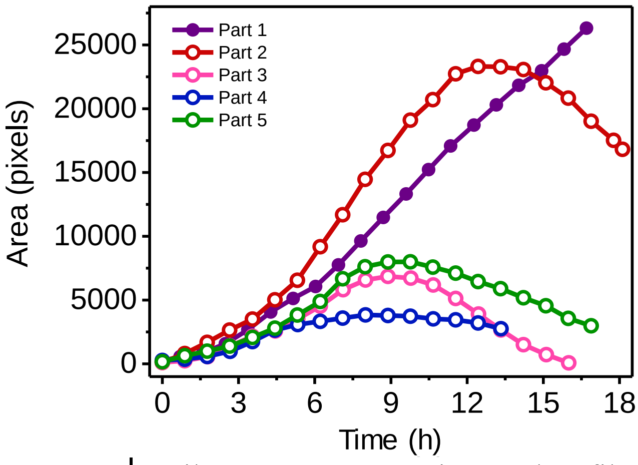

Supplementary Figure 25 │ Film area versus time. The films are highlighted in Supplementary Figure 24.

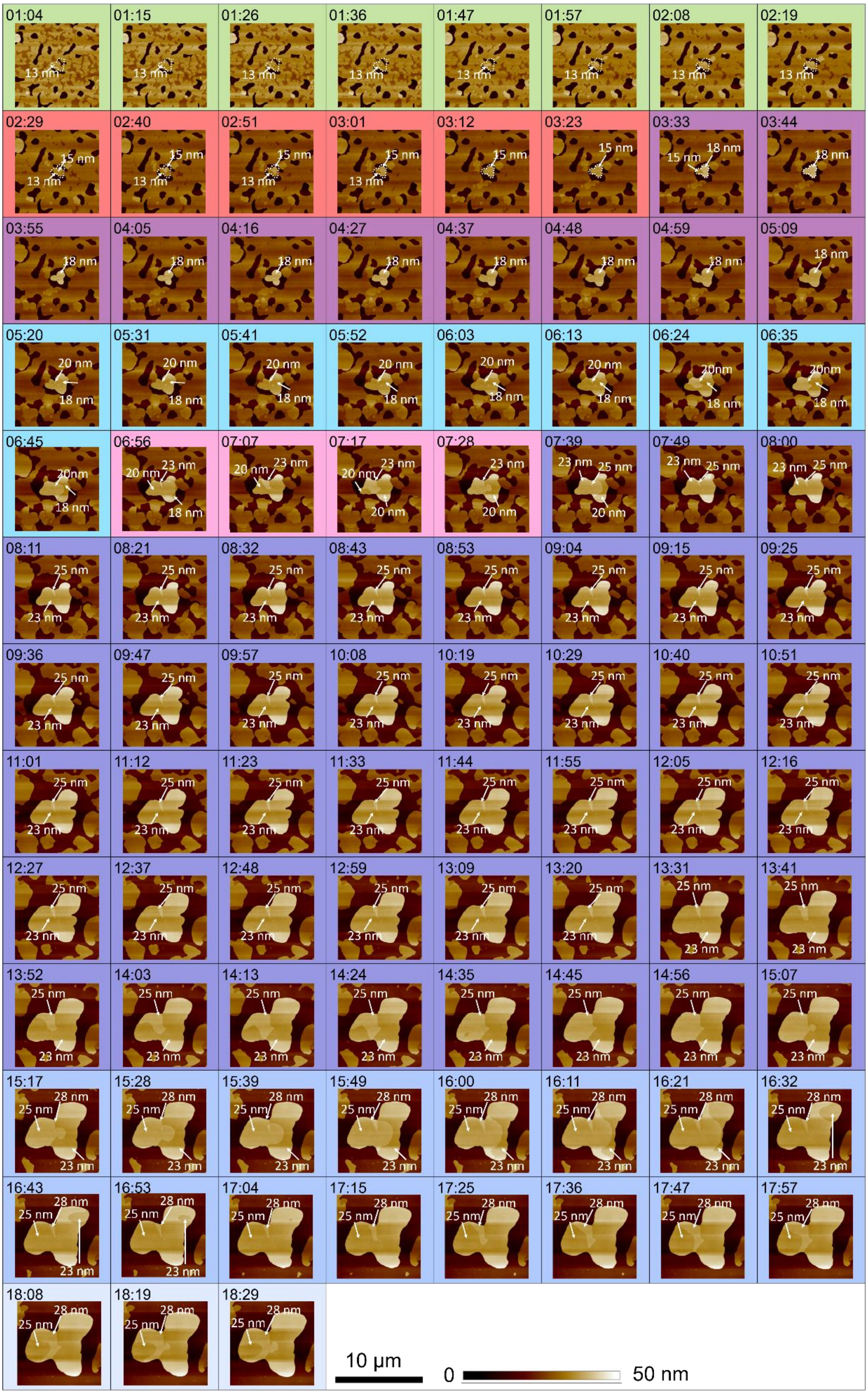

Supplementary Figure 26 | Time–lapse sequence of AFM images showing the self–organisation and layer growth mode (Sample 1).

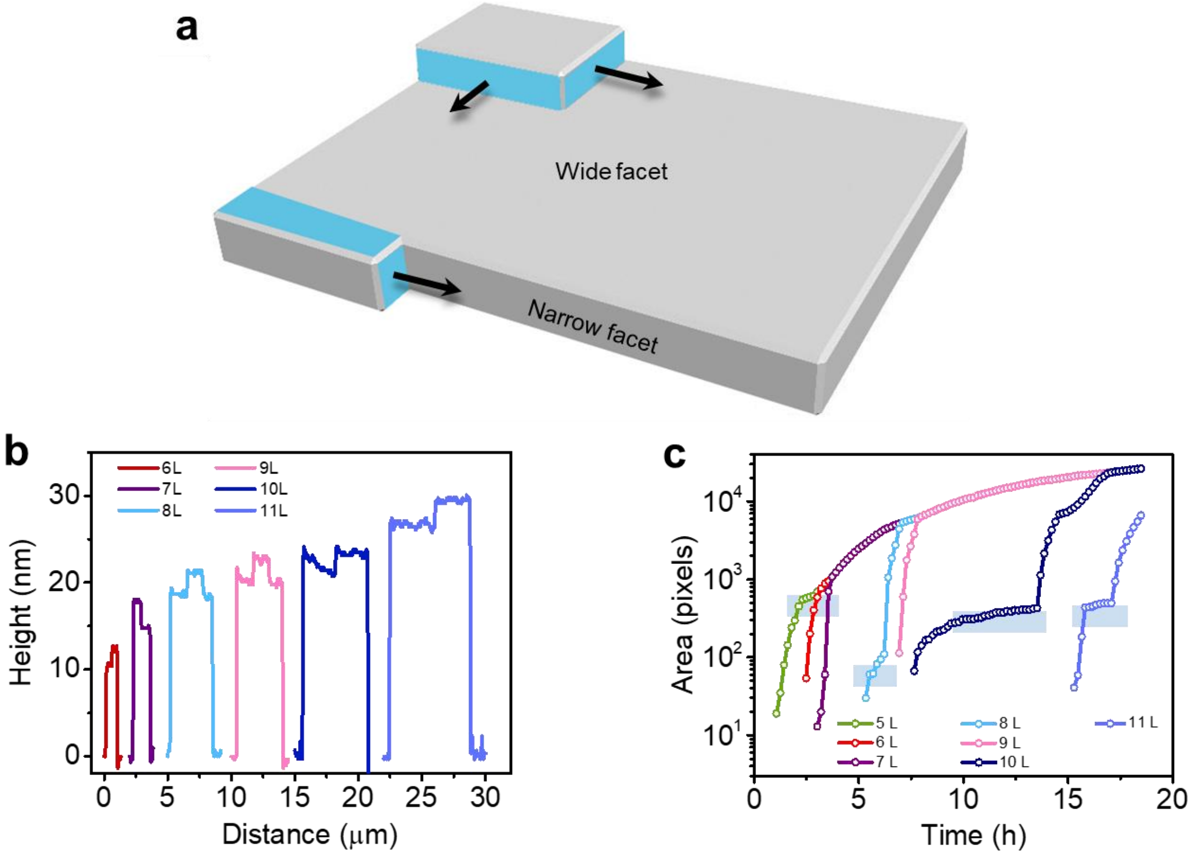

Supplementary Figure 27 | Theory of the layer growth mode. **a** Qualitatively different growth modes on wide and narrow facets of a single-crystal film. On large facets a nucleated island grows isotropically to minimise its energy. The corresponding nucleation barrier is determined by the critical island size. On narrow facets with thickness less than this critical size, the island quickly spans the entire facet and then grows along the facet. The corresponding nucleation barrier is substantially reduced, leading to the much faster growth at the experimental temperatures. **b** Height profiles of different layers (6 to 11 L) cross the edge from the substrate to each layer as shown in Supplementary Figure 26. The colors of the different lines in Supplementary Figure 27b correspond to different background colors in Supplementary Figure 26. **c** Film area versus time. The films are highlighted in different background colors in Supplementary Figure 26. We found that there was a period of relaxation after the nucleus generation, during which the new upper layers ceased to grow (light blue box).

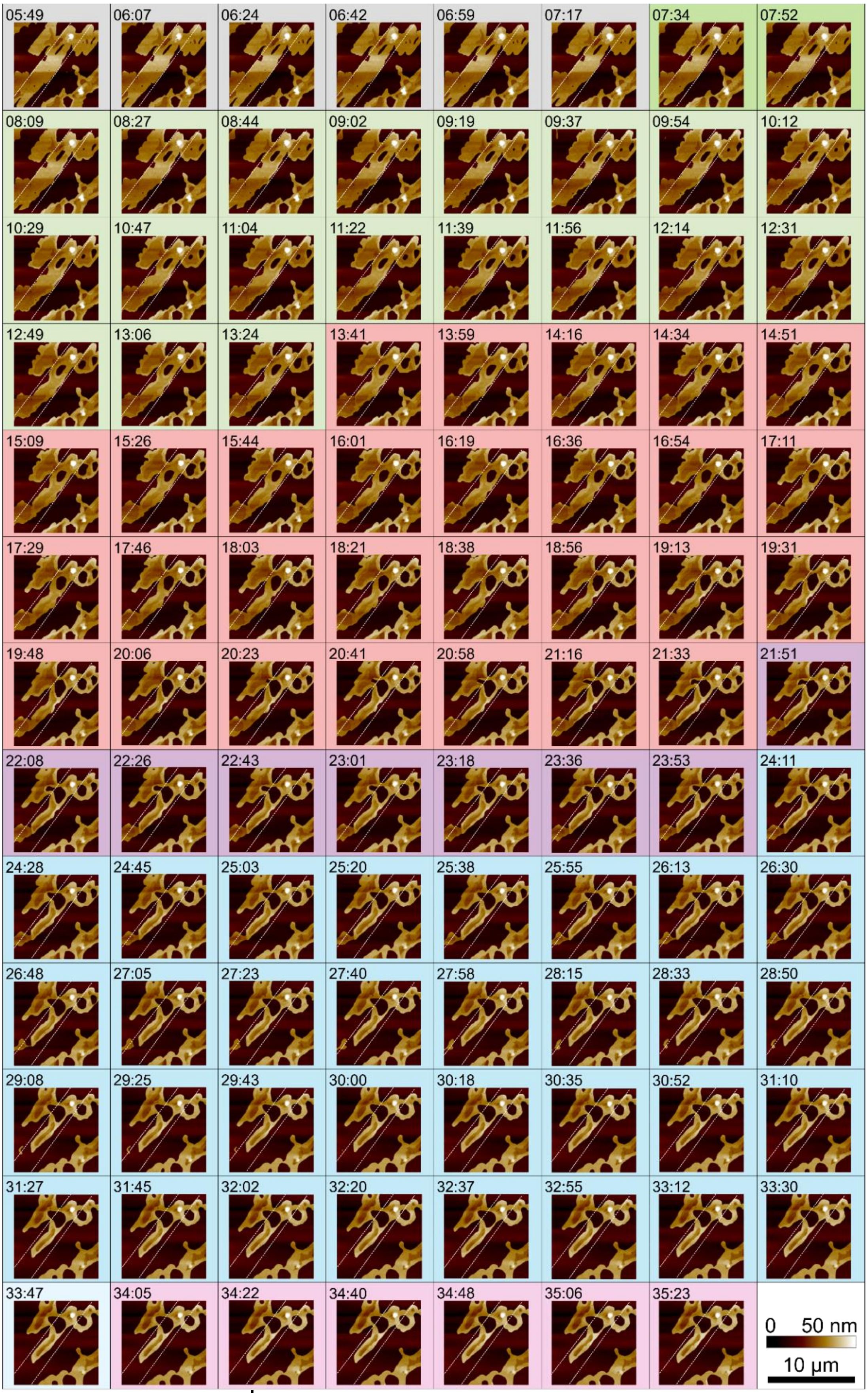

Supplementary Figure 28 | Time–lapse sequence of AFM images showing the self–confined layer growth mode (Sample 2).

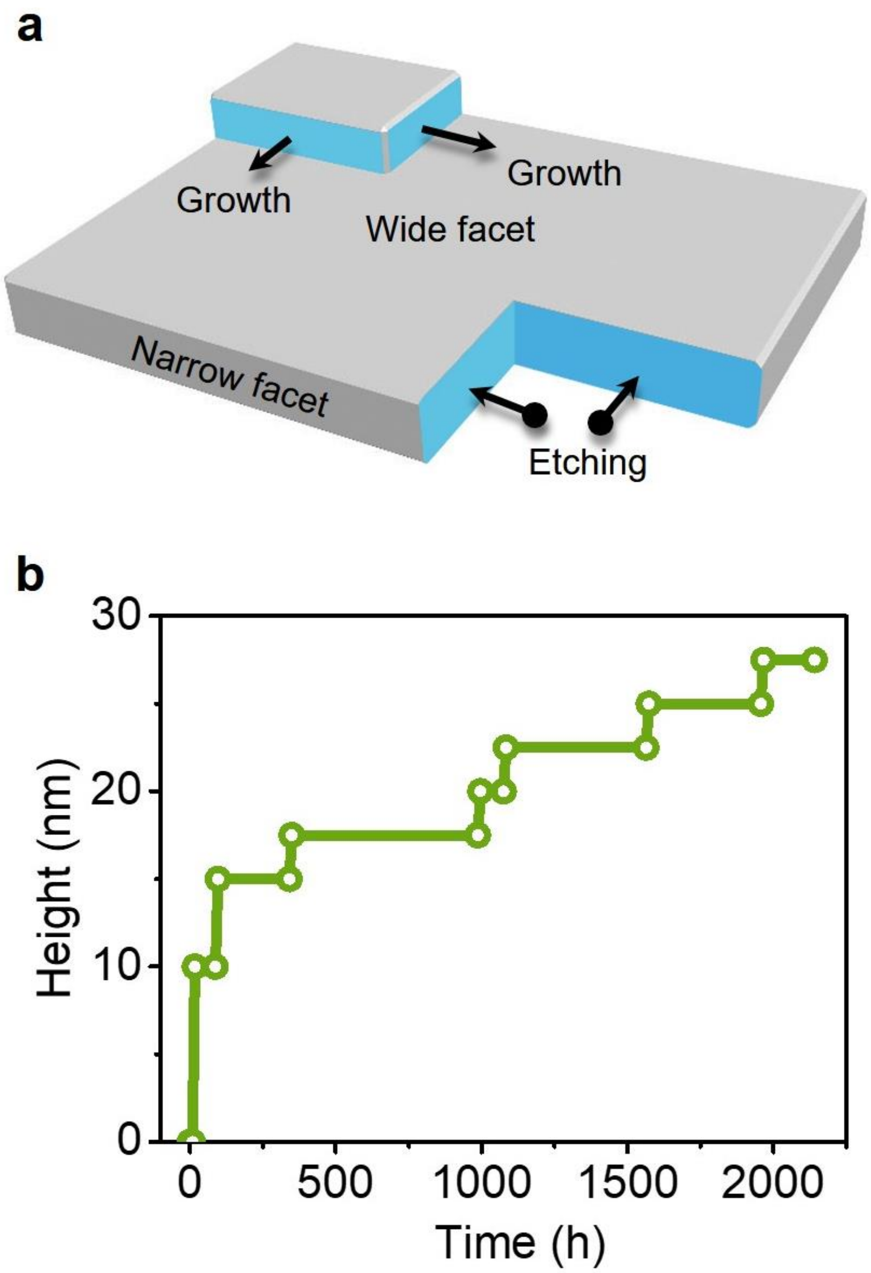

Supplementary Figure 29 │ Theory of the self-confined layer growth mode. **a** When the growing domain is totally isolated, it undergoes a self–confined layer growth mode, during which its high energy crystal facet is etched and serves as the molecular source to grow the upper layer. **b** Film height versus time. The films are highlighted in different background colors in Supplementary Figure 28.

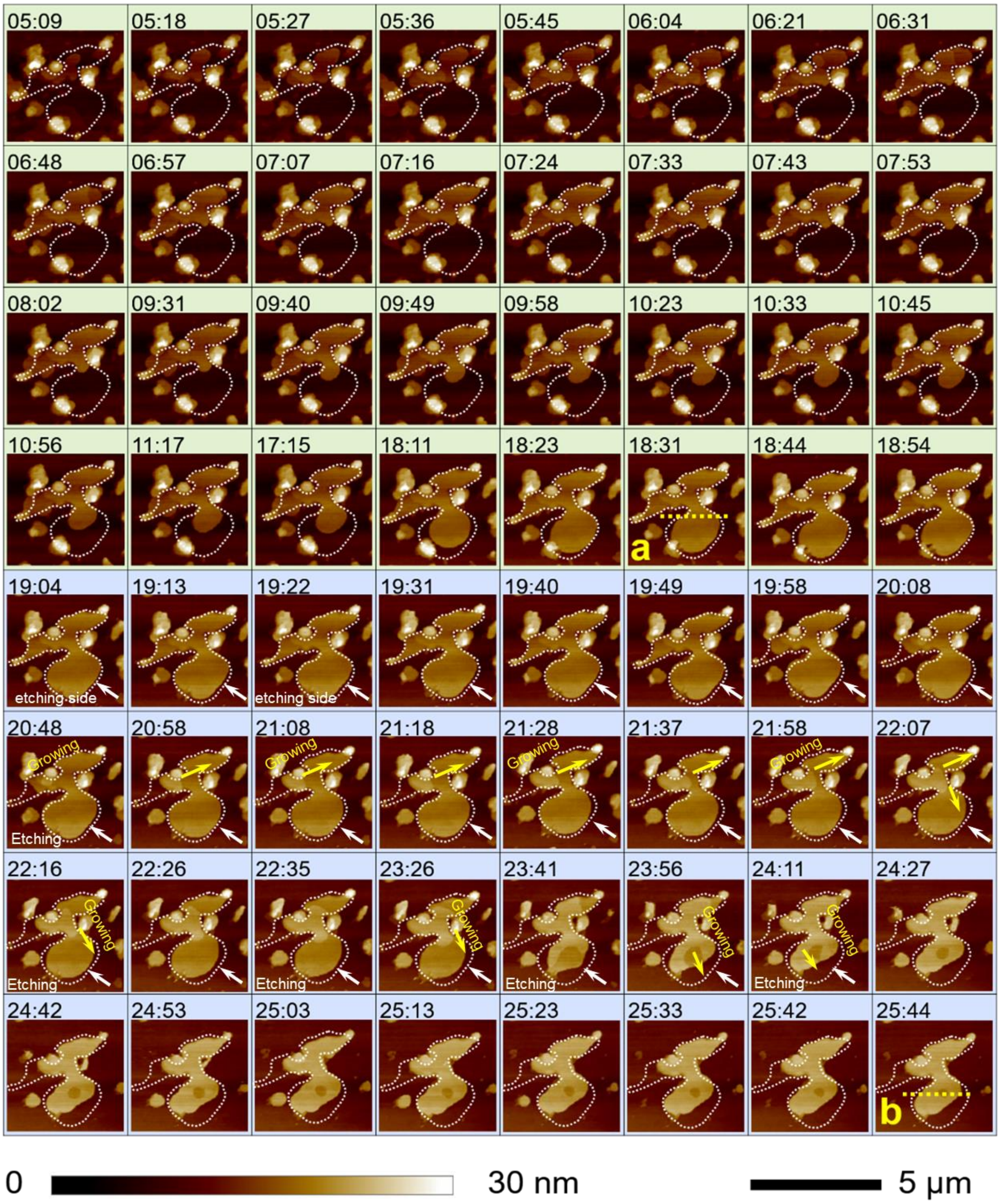

Supplementary Figure 30 | Time-lapse sequence of AFM images showing the self–confined layer growth mode (sample 3). The film grows continuously until t = 18 h 54 min (light green background). After that, etching starts from the lower right corner to grow the upper layer (light blue background). The white arrows indicate the etching direction of the 10-nm-thick layer while the yellow arrows indicate the growing direction of the 15–nm–thick layer.

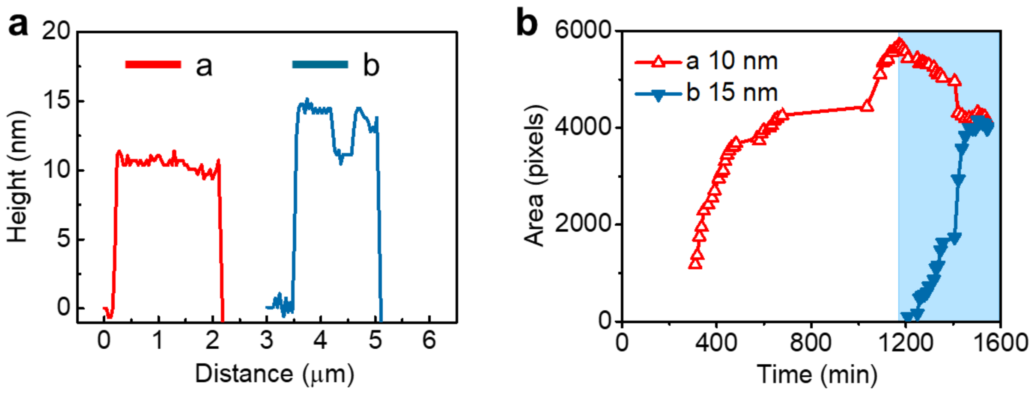

Supplementary Figure 31 │ Evolution statistics. **a** Height profile cross the edge from the substrate to each layer as shown in Supplementary Figure 30. **b** Film area versus time. The films are highlighted in Supplementary Figure 30.

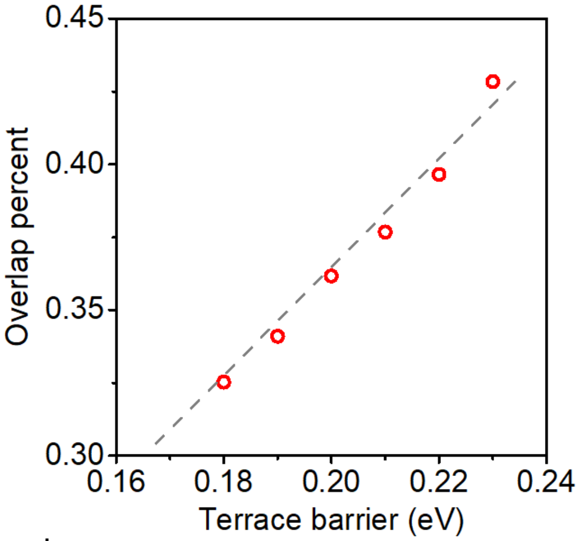

Supplementary Figure 32 │ Overlap percent under different terrace barriers (The detachment barrier = 0.25 eV).

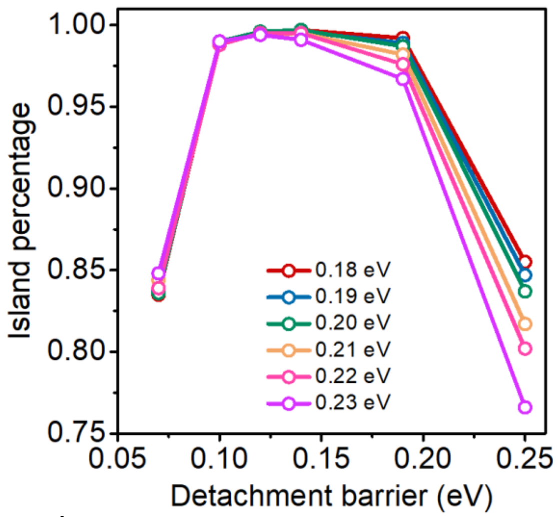

Supplementary Figure 33 │ Island percentage versus detachment barrier under different terrace barriers.

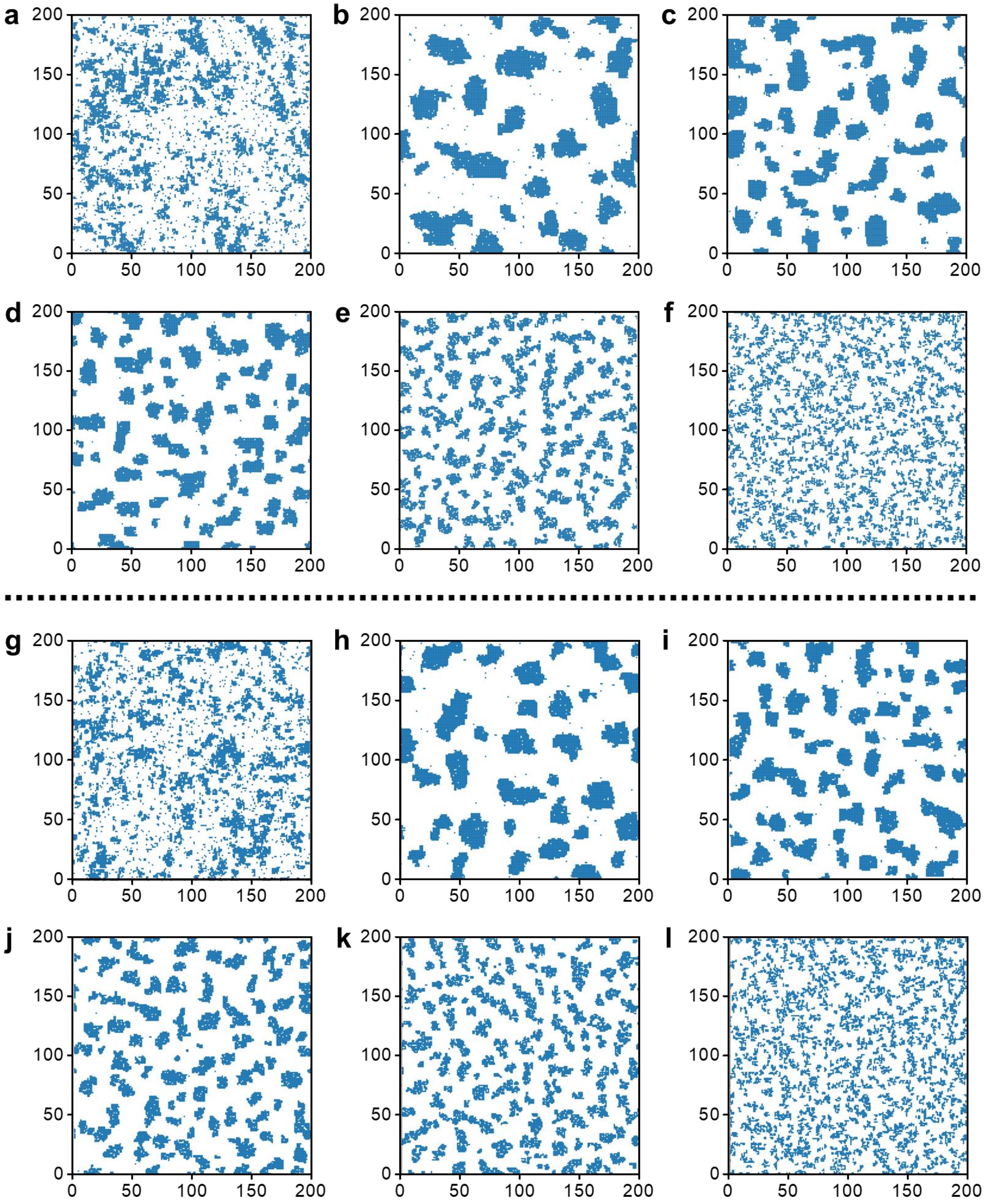

Supplementary Figure 34 | ** Total effect. **a–**f** Island morphologies in the same time when fixing the detachment barrier while increasing the terrace barrier from 0.07 eV (**a**), 0.10 eV (**b**), 0.12 eV (**c**), 0.14 eV (**d**), 0.19 eV (**e**), to 0.25 eV (**f**). **g**–**l** Island morphologies in the same time when fixing the terrace barrier while increasing the detachment barrier from 0.07 eV (**g**), 0.10 eV (**h**), 0.12 eV (**i**), 0.14 eV (**j**), 0.19 eV (**k**), to 0.25 eV (**l**).

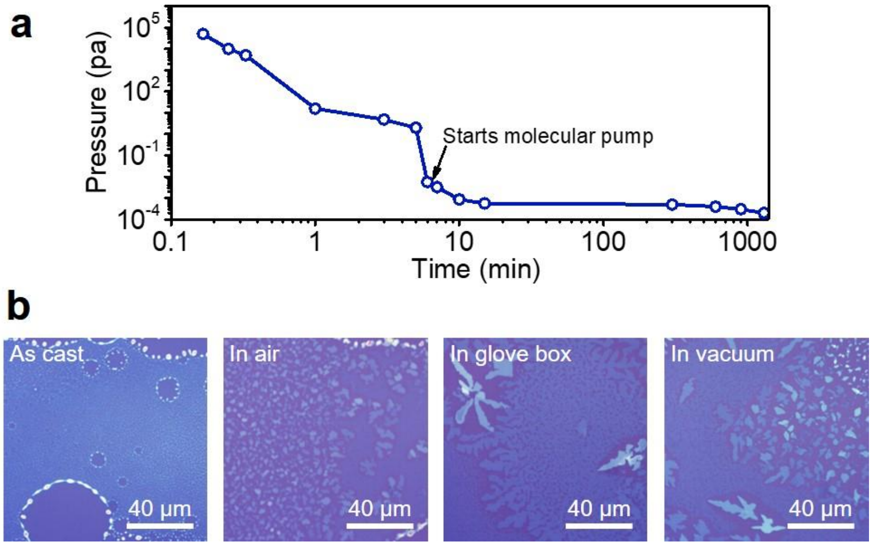

Supplementary Figure 35 │ Exclusion of the effect of solvent. **a** Pressure of the chamber where the wafer was put in for 24 h. **b** OM images showing the morphology of films kept in air, glove box, and vacuum, respectively.

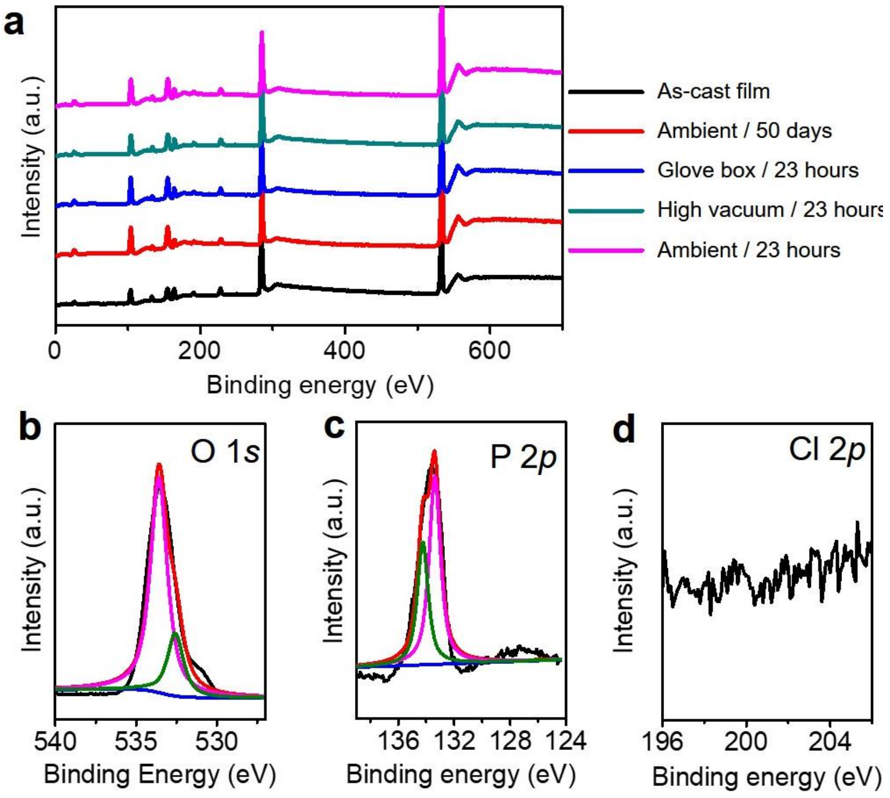

Supplementary Figure 36 │ XPS characterisation of as-prepared thin-films. **a** XPS survey scan of thin films under different preservation conditions. **b–d** High-resolution XPS for O 1*s* (**b**), P 2*p* (**c**), and Cl 2*p* (**d**). The appearance of peaks from P revealed the formation of C_7P–BTBT thin films on the SiO_2 surface. No Cl signals proved the absence of $CHCl_3$ solvent residuals.

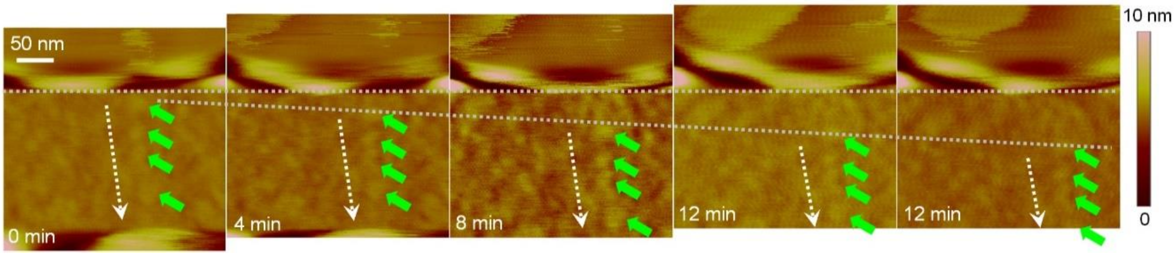

Supplementary Figure 37 │ AFM images showing the spherical molecular cluster as mass transport carriers between two domains on surface at room temperature (Green arrows marked four nanoclusters; White dash arrows marked the moving directions; Grey dash lines are the leading lines).

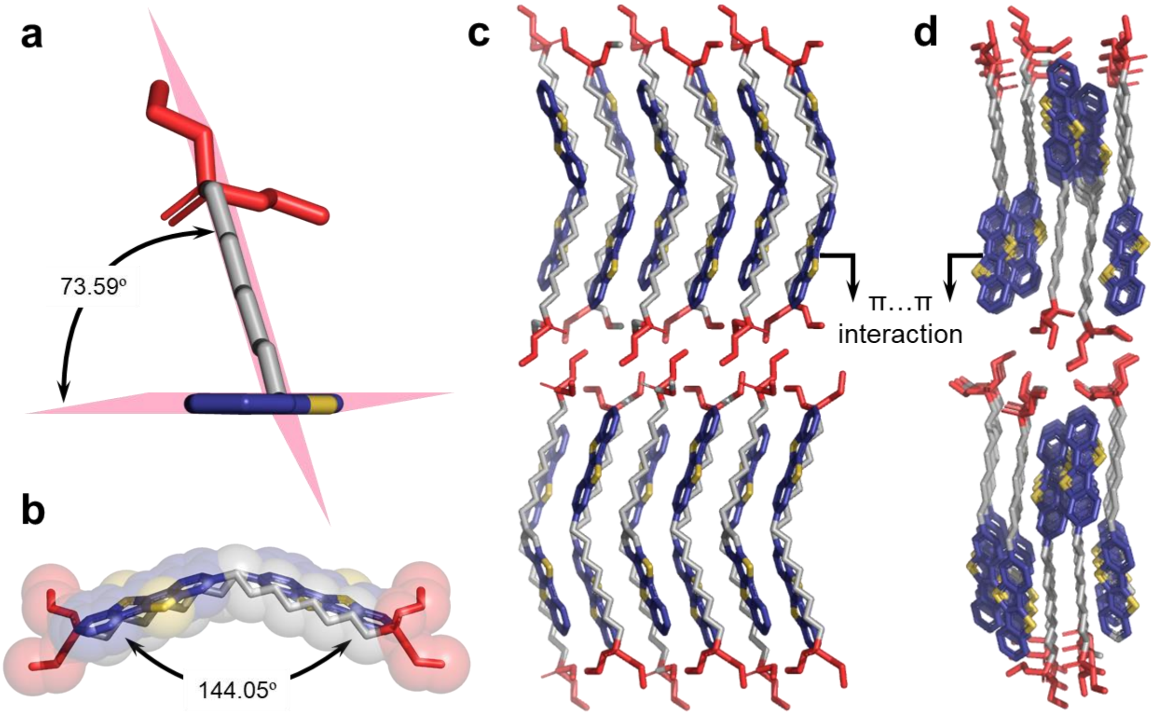

Supplementary Figure 38 │ Solid–state (super)structures of kinked C_8P-BTBT obtained from X-ray crystallography on single crystals. **a,** Stick representation of C_8P-BTBT showing that the dihedral angle between BTBT core and alkyl phosphonate coronas is ~73.59º. **b,** The induced kink angel between the BTBT core and alkyl phosphonate coronas is ~144.05º. **c,** Superstructure of the kinked C_8P-BTBT revealing a bowl shaped packing. **d,** Perspective view of the superstructure revealing the π-π interaction of the BTBT core.

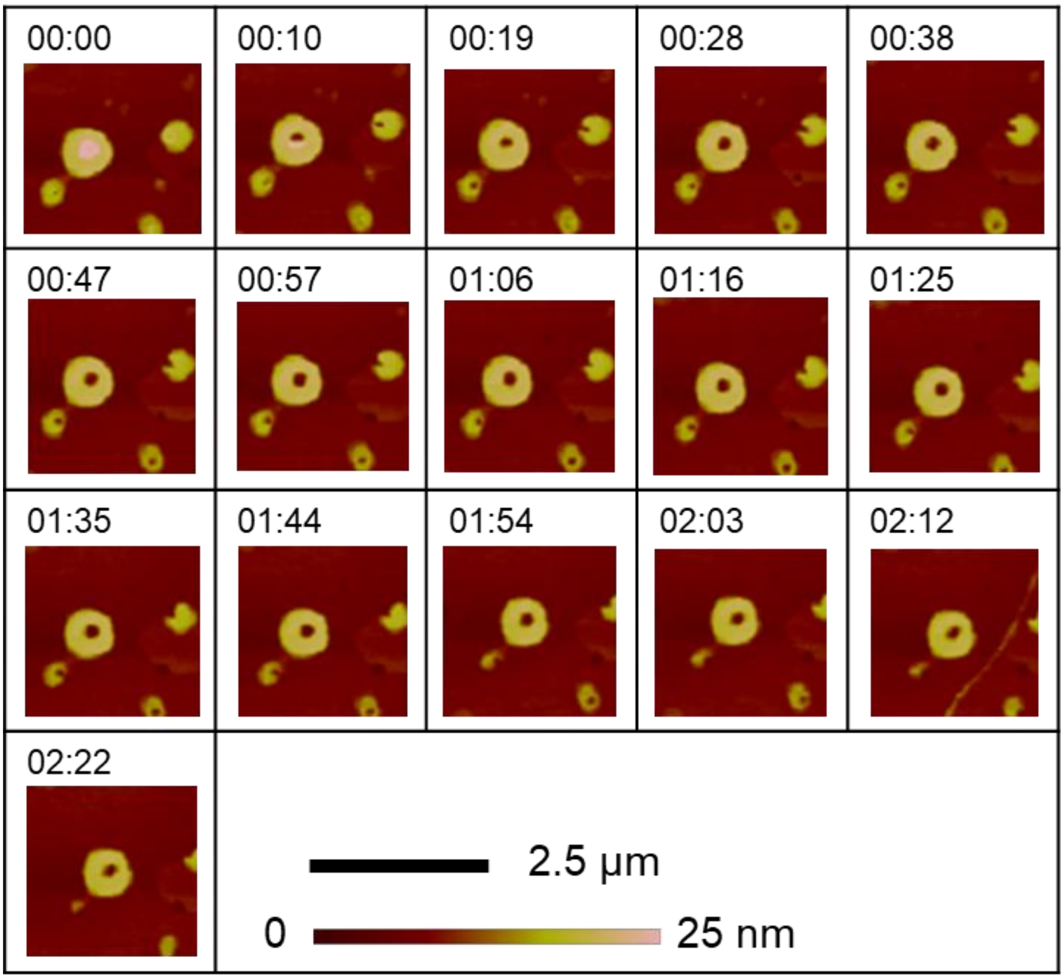

Supplementary Figure 39 | Film area evolution of C_5P–BTBT on SiO_2 substrates.

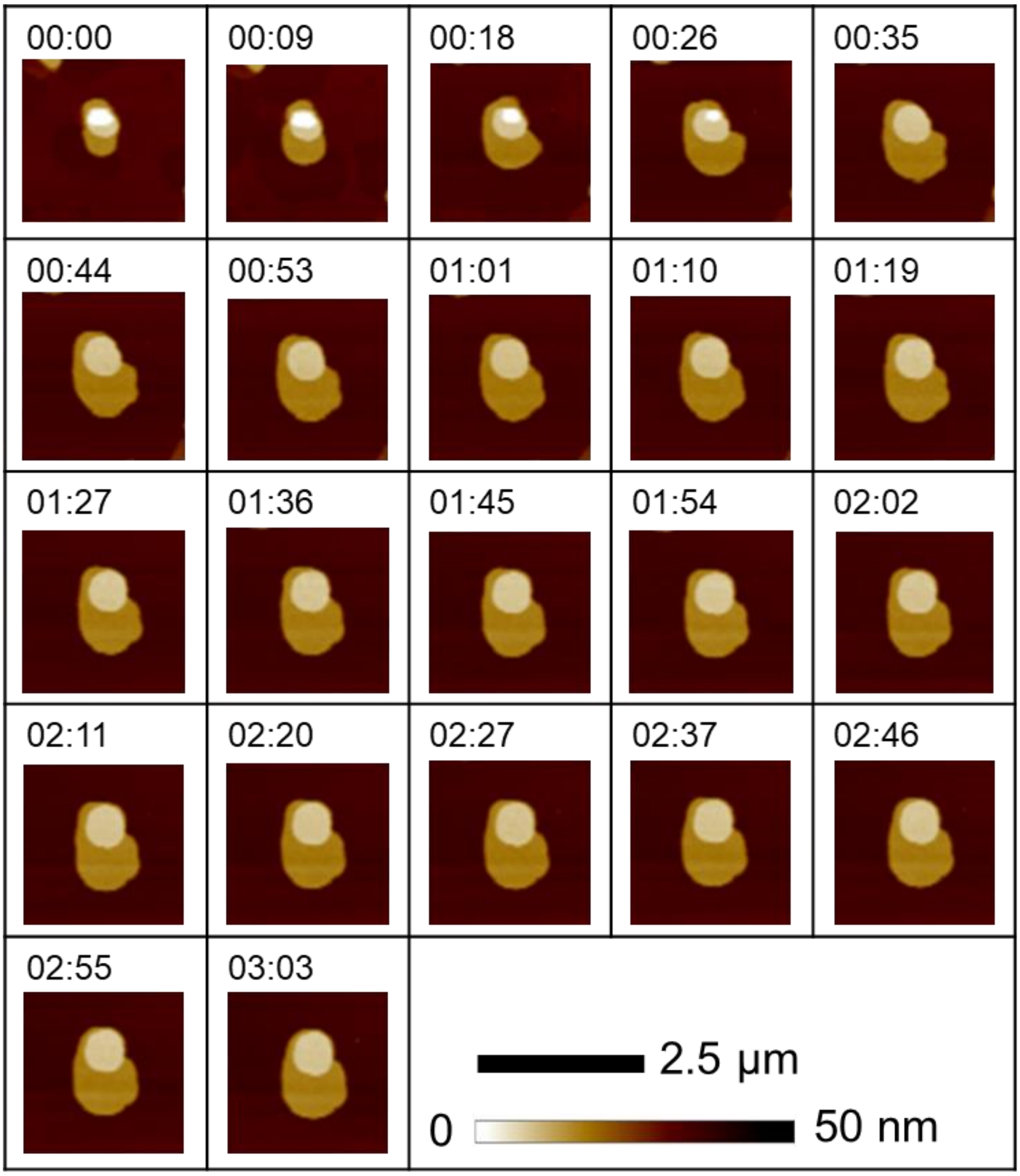

Supplementary Figure 40 │ Film area evolution of C_6P–BTBT on SiO_2 substrates.

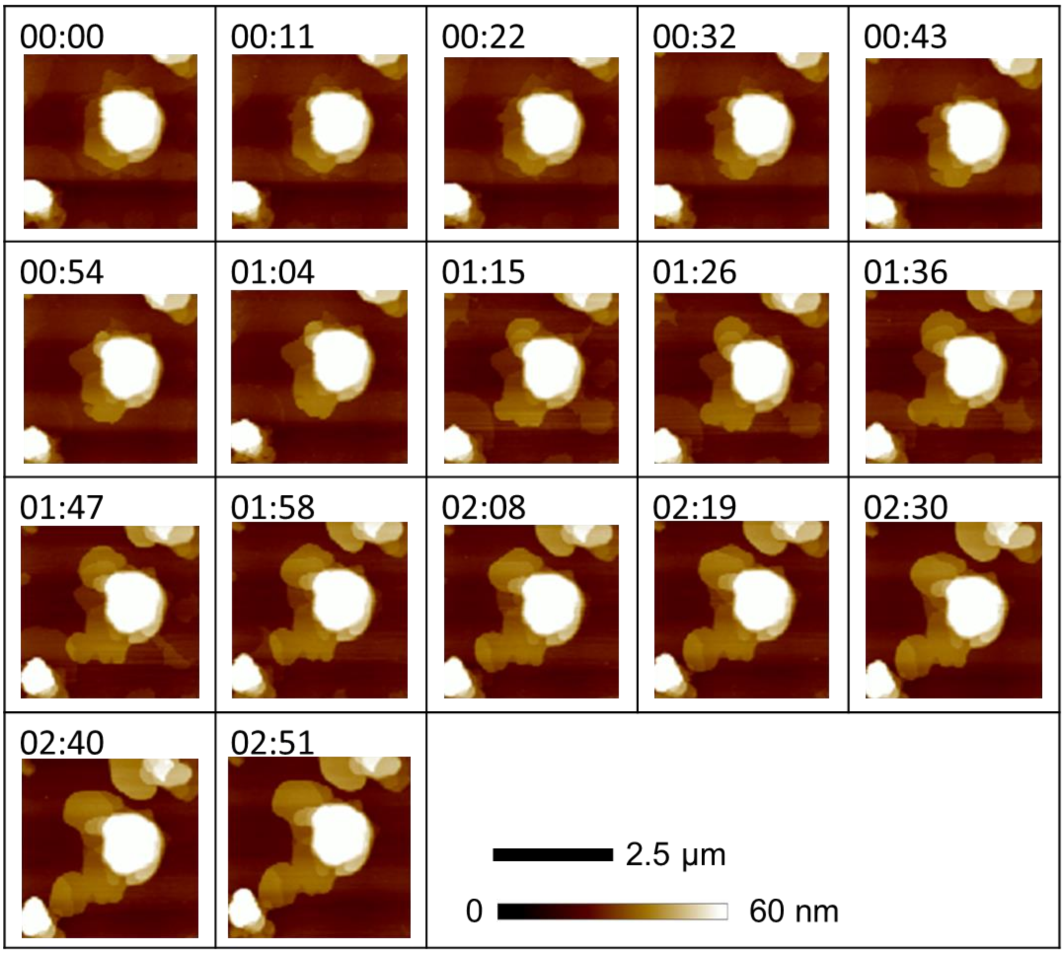

Supplementary Figure 41 | Film area evolution of C_8P–BTBT on SiO_2 substrates.

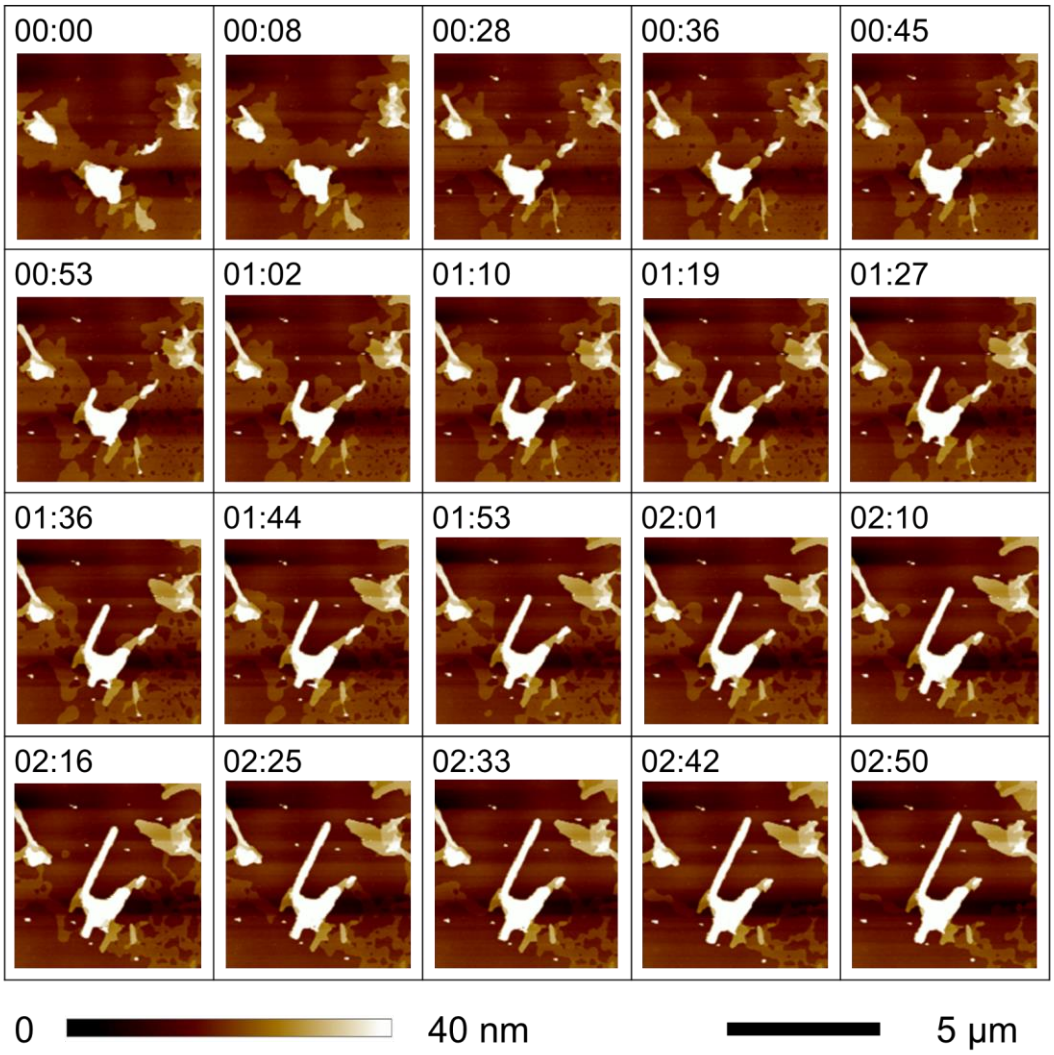

Supplementary Figure 42 | Film area evolution of C₉P–BTBT on SiO₂ substrates.

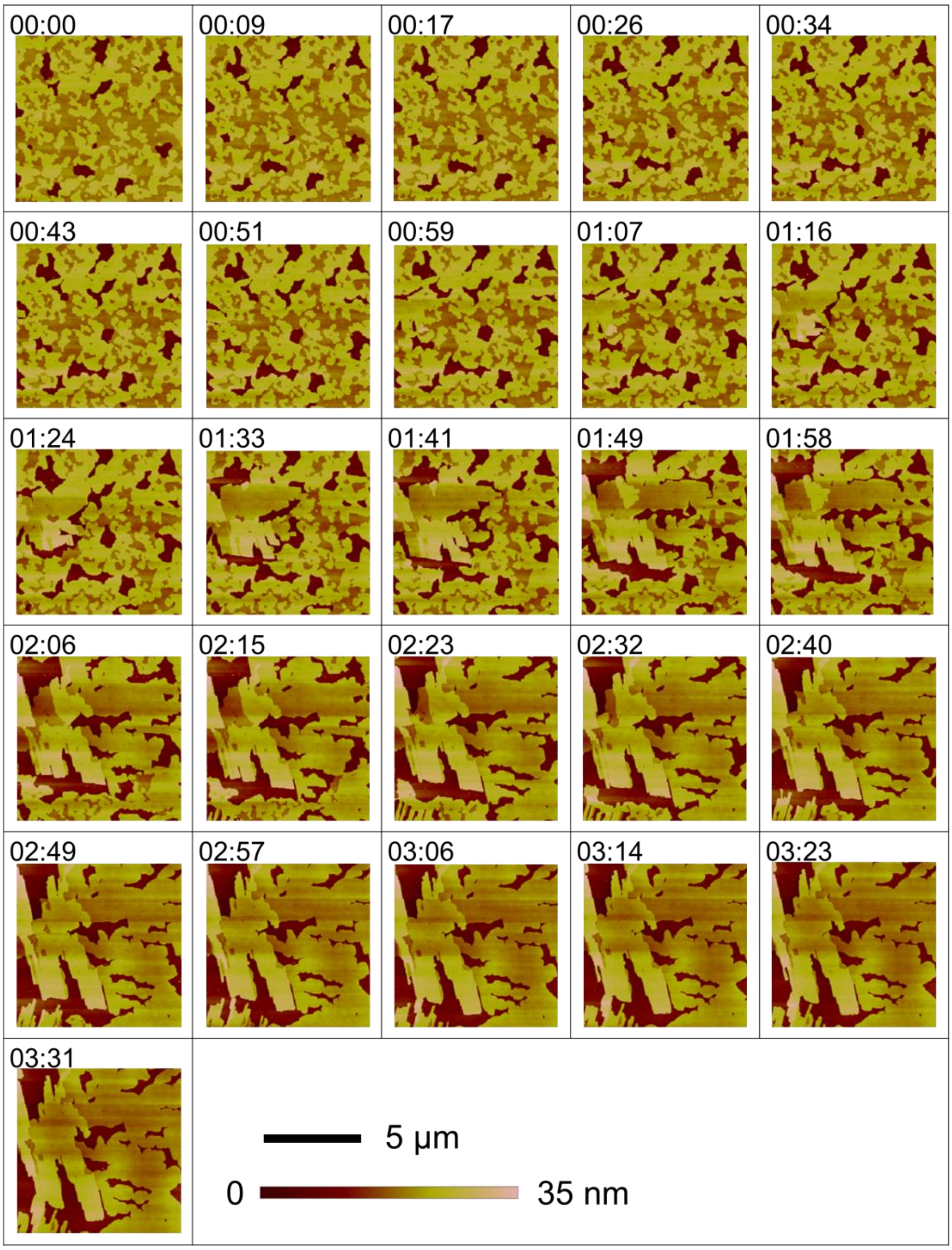

Supplementary Figure 43 │ Film area evolution of C_{10}P–BTBT on SiO_2 substrates.

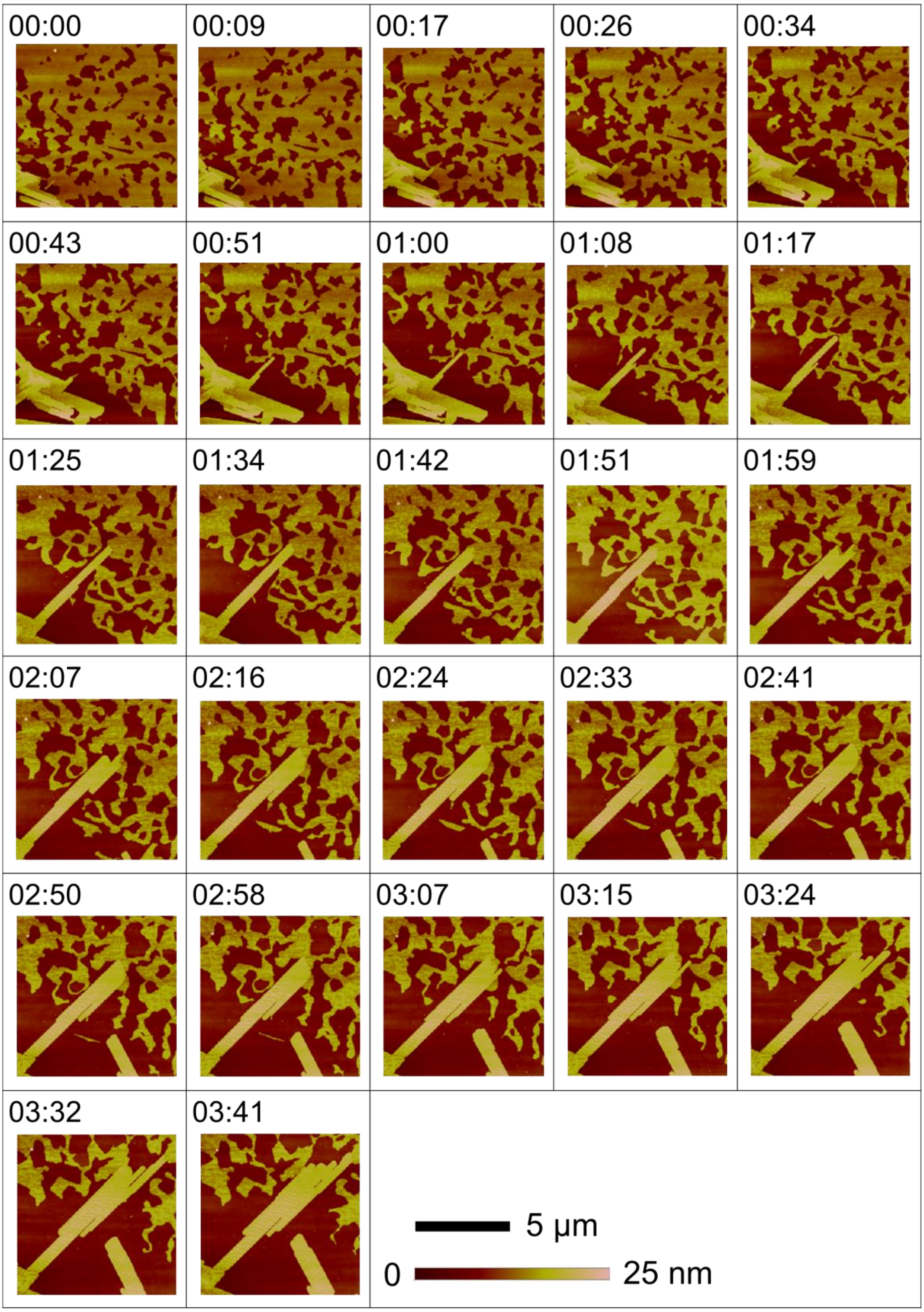

Supplementary Figure 44 │ Film area evolution of C_{11}P–BTBT on SiO_2 substrates.

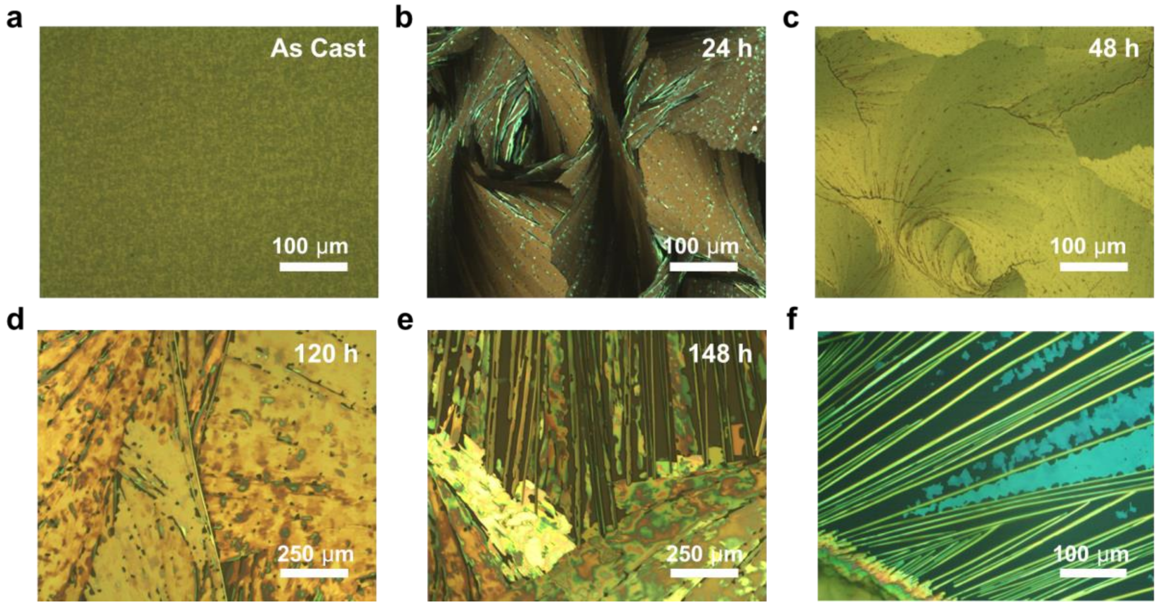

Supplementary Figure 45 │ OM images showing the single crystal microwire (MW) cyrstallisation from solid films. Time-lapse sequence of OM images taken at as-cast (**a**), 24 h (**b**), 48 h (**c**), 120 h (**d**), and148 h (**e**). (**f**) A magnified image of (**e**).

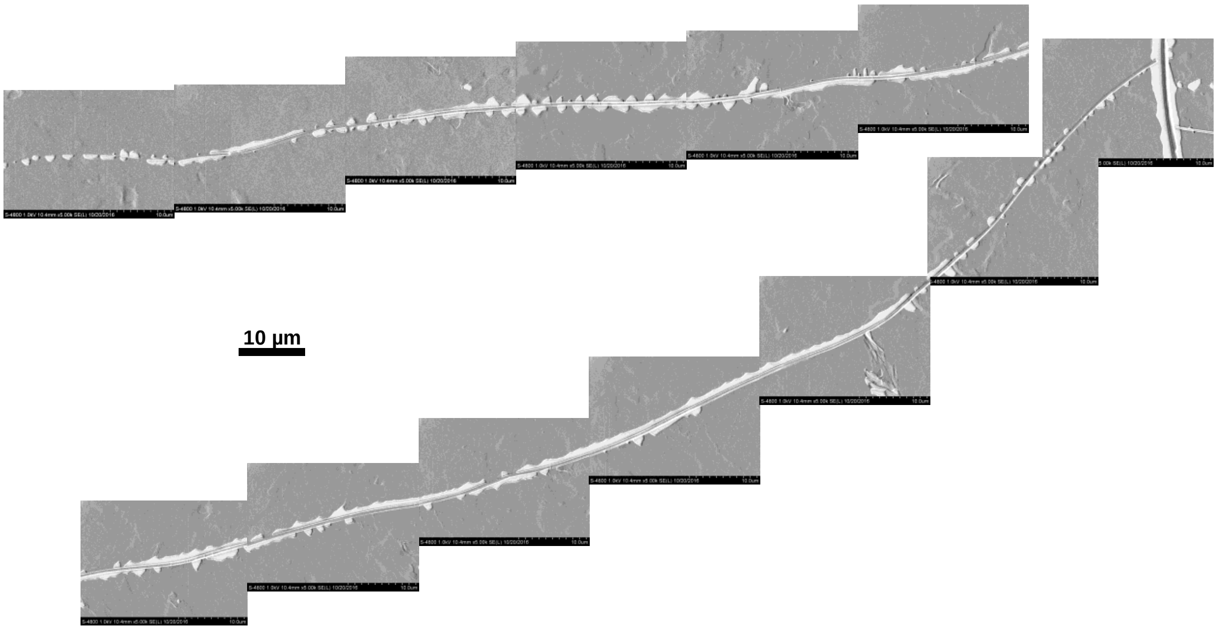

Supplementary Figure 46 │ SEM images showing the formation of ultralong (> 300 μm) microwires from solid films.

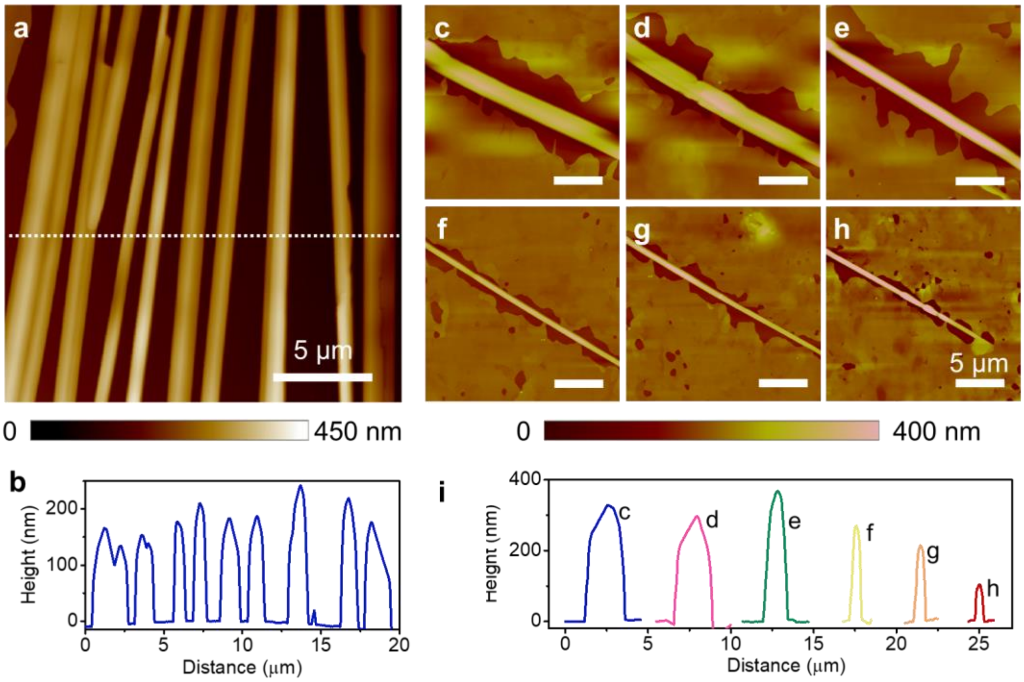

Supplementary Figure 47 │ AFM characterisations of MWs. **a,** AFM image showing morphologies of C_7P-BTBT MWs. **b,** The crosponding hight profile cross the edge from the substrate to each MW as shown in Fig. 47a. **c–h,** AFM images showing morphologies of MWs with the different growing times. **i,** The crosponding hight profile of MWs from **c** to **h**.

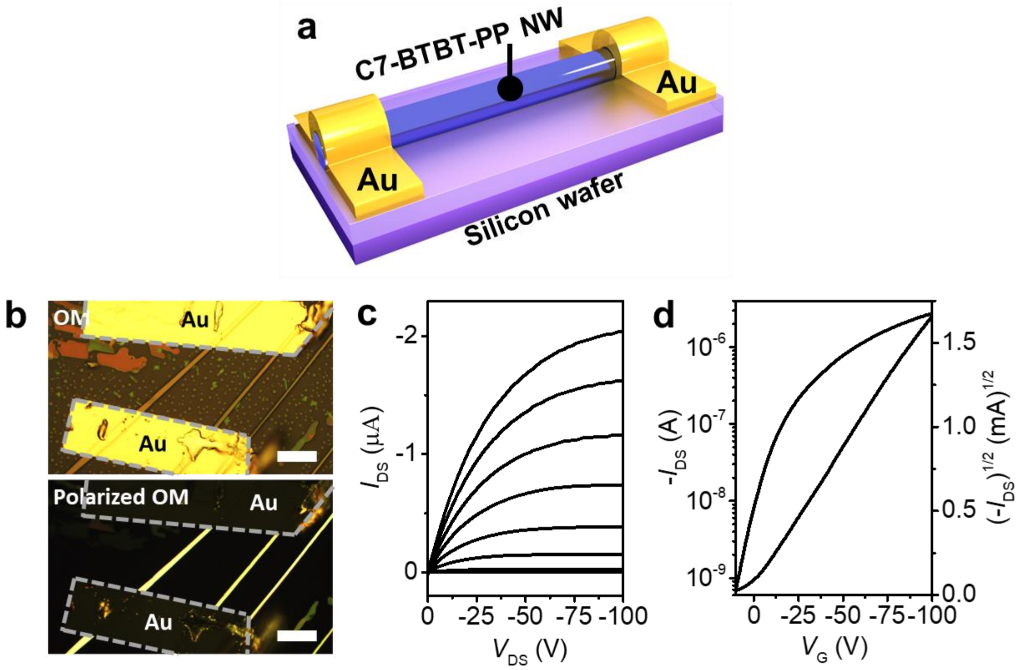

Supplementary Figure 48 │ Electrical performance characterisation. **a,** Diagram of a BG/TC OFET device. **b,** OM (top) and polarised OM (bottom) images of a fabricated OFET device. L = 30 and W = 1.5 μm. Scale bar: 10 μm. **c,** Output characteristics of a microwire single crystal OFET with the gate voltage (V_{GS}) ranged from 10 to –95 V in –15 V steps. **d,** Transfer characteristics with the source-drain voltage (V_{DS}) = –50 V.

Supplementary Tables

Supplementary Table 1 │ Energy band gap (Δ*E*) obtained from UV–Vis absorption spectra.

No.	Solution		Thin film	
	λ (nm)	Δ*E* (eV)	*λ* (nm)	Δ*E* (eV)
**3**	340	3.65	368	3.37
**4**	339	3.66	370	3.35
**5**	339	3.66	370	3.35
**6**	340	3.65	353	3.51
**7**	342	3.63	350	3.54
**8**	340	3.65	350	3.54
**9**	348	3.56	372	3.33
**10**	340	3.65	3.58	3.46
**11**	340	3.65	3.70	3.35

Supplementary Table 2 │ Energy levels of C_nP-BTBT (n = 3 to 11) obtained from CV measurements.

No.	Solution			Thin film		
	HOMO (eV)	LUMO (eV)	Gap (eV)	HOMO (eV)	LUMO (eV)	Gap (eV)
**3**	–5.738	–1.906	3.832	–5.778	–1.940	3.838
**4**	–5.773	–1.887	3.886	–5.783	–1.926	3.857
**5**	–5.748	–1.897	3.851	–5.765	–1.890	3.875
**6**	–5.753	–1.881	3.872	–5.759	–1.858	3.901
**7**	–5.736	–1.963	3.773	–5.671	–1.909	3.762
**8**	–5.754	–1.898	3.856	–5.725	–1.925	3.800
**9**	–5.591	–1.879	3.712	–5.684	–1.854	3.830
**10**	–5.724	–1.872	3.852	–5.715	–1.915	3.800
**11**	–5.761	–1.892	3.869	–5.861	–1.903	3.958

Supplementary Table 3 | Summary of the phase transition results.

	Heating			Cooling		
Molecular Number (n)	Phase Transition[a)]	T_{trans} (℃)	△H (kJ/mol)	Phase Transition[a)]	T_{trans} (℃)	△H (kJ/mol)
3	$Cr_{stable} \rightarrow Cr_1$	93.01	1.1			
	$Cr_1 \rightarrow Cr_2$	94.02	-1.9			
	$Cr_2 \rightarrow IL$	101.31	23.5			
	$Cr \rightarrow IL$	57.76	9.7	$IL \rightarrow Cr$	50.81	–9.7
4	$Cr_{stable} \rightarrow IL$	59.32	21.1			
	$Cr \rightarrow Cr_{meta}$	32.67	1.1			
	$Cr_{meta} \rightarrow IL$	59.47	19.5			
				$IL \rightarrow LC$	29.67	–5.5
				$LC \rightarrow Cr$	5.83	–3.5
5	$Cr_{stable} \rightarrow IL$	52.77	8.8			
	$Cr \rightarrow IL$	46.27	7.5	$IL \rightarrow Cr$	39.66	–7.8
6	$Cr_{stable} \rightarrow Cr1$	13.88	6.4			
	$Cr_1 \rightarrow IL$	71.07	18.0			
	$Cr \rightarrow LC$	29.64	3.6	$LC \rightarrow Cr$	20.61	–3.3
	$LC \rightarrow IL$	65.67	15.8	$IL \rightarrow LC$	53.67	–14.9
7	$Cr_{stable} \rightarrow IL$	61.14	43.1			
	$Cr \rightarrow IL$	57.75	28.5	$IL \rightarrow Cr$	44.03	–28.1
8	$Cr_{stable} \rightarrow Cr1$	52.59	0.3			
	$Cr_1 \rightarrow Cr_2$	58.85	0.6			
	$Cr_2 \rightarrow Cr_3$	75.23	32.7			
	$Cr_3 \rightarrow IL$	77.32				
	$Cr \rightarrow IL$	77.04	32.0	$IL \rightarrow Cr$	52.34	–30.1
9	$Cr_{stable} \rightarrow IL$	67.80	41.4			
	$Cr \rightarrow IL$	67.80	40.5			
				$IL \rightarrow LC$	59.10	–7.2
				$LC \rightarrow Cr$	36.53	–22.4
10	$Cr_{stable} \rightarrow IL$	66.82	48.6			
	$Cr \rightarrow Cr_{meta}$	38.51	9.5			
	$Cr_{meta} \rightarrow IL$	66.31	36.5			
				$IL \rightarrow LC$	57.23	–8.3
				$LC \rightarrow Cr_1$	35.99	–15.7
				$Cr_1 \rightarrow Cr$	32.23	
11	$Cr_{stable} \rightarrow IL$	61.52	46.9			
	$Cr \rightarrow Cr_{meta}$	44.43	6.0			
	$Cr_{meta} \rightarrow IL$	61.47	45.2			
				$IL \rightarrow LC$	55.74	–9.4
				$LC \rightarrow Cr$	31.35	–20.8

a) Cr_{stable}: Stable polymorphic form after a storage; Cr: Crystal Phase; Cr_1: Crystal phase 1; Cr_2: Crystal phase 2; Cr_3: Crystal phase 3; IL: Isotropic liquid phase; LC: Liquid crystal phase; Cr_{meta}: metastable polymorphic form which converts into the stable crystal phase.

Supplementary Table 4 │ Summary of Crystallographic data.

Molecule		C_3P–BTBT	C_4P–BTBT	C_5P–BTBT	C_6P–BTBT	C_7P–BTBT	C_8P–BTBT	C_9P–BTBT	$C_{10}P$–BTBT	$C_{11}P$–BTBT
Moiety formula		$C_{21}H_{23}O_3PS_2$	$C_{22}H_{25}O_3PS_2$	$C_{23}H_{27}O_3PS_2$	$C_{24}H_{29}O_3PS_2$	$C_{25}H_{31}O_3PS_2$	$2(C_{26}H_{33}O_3PS_2)$, H_2O	$C_{27}H_{35}O_3PS_2$	$C_{29}H_{37}O_3PS_2$	$C_{30}H_{39}O_3PS_2$
Sum formula		$C_{21}H_{23}O_3PS_2$	$C_{22}H_{25}O_3PS_2$	$C_{23}H_{27}O_3PS_2$	$C_{24}H_{29}O_3PS_2$	$C_{25}H_{31}O_3PS_2$	$C_{52}H_{68}O_7P_2S_4$	$C_{27}H_{35}O_3PS_2$	$C_{29}H_{37}O_3PS_2$	$C_{30}H_{39}O_3PS_2$
Molecular weight		418.48	432.51	446.54	460.56	474.59	995.24	502.64	516.67	530.69
Crystal system		Triclinic	Monoclinic	Monoclinic	Triclinic	Monoclinic	Monoclinic	Monoclinic	orthorhombic	Monoclinic
Space group		*P-1*	$P\,2_1$	$P\,2_1/c$	*P-1*	$P\,2_1/c$	$P\,2_1/c$	$P\,2_1/c$	*P bcn*	$P\,2_1/c$
a (Å)		8.1396(3)	8.1566(8)	18.8298(8)	8.0619(2)	11.5360(4)	8.2750(2)	30.927(2)	14.7879(5)	33.1999(19)
b (Å)		8.6058(3)	5.9447(4)	48.168(2)	8.7602(2)	51.8770(15)	53.407(3)	14.8221(10)	5.6793(2)	15.0627(8)
c (Å)		29.5204(11)	22.882(3)	7.3938(5)	16.4641(3)	7.2120(6)	10.6370(2)	5.7635(4)	63.721(2)	5.6030(3)
α (°)		90.683(3)	90.00	90.00	92.538(2)	90.00	90.00	90.00	90.00	90.00
β (°)		96.590(3)	99.633(11)	96.264(5)	94.731(2)	105.565(5)	109.775(3)	91.733(6)	90.00	91.019(5)
γ (°)		93.764(3)	90.00	90.00	92.226(2)	90.00	90.00	90.00	90.00	90.00
Volume (Å^3)		2049.34(14)	1093.87(18)	6666.1(6)	1156.60(4)	4157.8(4)	4423.7(3)	2640.8(3)	5351.6(3)	2801.5(3)
Z		4	2	12	2	8	4	4	8	4
Temperature (K)		180.0(1)	180.0(1)	180.0(1)	180.0(1)	100(1)	100(1)	180.0(1)	180.0(1)	180.0(1)
Theta range		3.53° < θ < 26.37°	3.54° < θ < 26.37°	3.44° < θ < 26.37°	3.49° < θ < 26.37°	1.44° < θ < 23.75°	1.40° < θ < 24.07°	3.79° < θ < 26.37°	3.48° < θ < 26.37°	3.65° < θ < 26.37°
Reflection collected		19614	12256	31243	12924	8174	8430	15665	40312	29906
Unique reflection		8316	4366	13389	4673	8174	8430	5320	5440	5687
Data completeness		99.1%	99.2%	98.2%	99.0%	99.7%	93.0%	98.4%	99.6%	99.7%
Goodness-of-fit on F^2		0.938	1.010	1.023	1.075	1.001	1.079	1.189	1.144	1.140
Final R indices [$I > 2\sigma(I)$]	$R1$	0.0794	0.0710	0.1438	0.0336	0.0472	0.0826	0.0819	0.0940	0.0680
	$wR2$	0.1762	0.1604	0.3489	0.0922	0.1231	0.1885	0.1776	0.1812	0.0825
R indices (all data)	$R1$	0.0943	0.1196	0.2125	0.0412	0.0562	0.1136	0.1008	0.1087	0.1422
	$wR2$	0.1861	0.1807	0.3825	0.0963	0.1305	0.2085	0.1842	0.1864	0.1497
Largest difference peak or hole (e. Å^{-3})		0.694 and –0.698	0.655 and –0.419	1.264 and –1.021	0.514 and –0.328	0.767 and –0.472	1.385 and –0.719	0.601 and –0.412	0.731 and –0.703	0.714 and –0.447

More explanations for the alerts appeared in the check-cif files.

Alerts	Explanations
The structure of C_8P–BTBT, Alert level A: PLAT029_ALERT_3_A _diffrn_measured_fraction_theta_full value Low	The data completeness for C_8P–BTBT is a little low (93.0%), which resulted from the limited rotation range of the sample in Synchrotron single crystal X-ray diffraction experiments.
The structures of C_3P–BTBT, C_5P–BTBT, C_6P–BTBT, C_9P–BTBT, $C_{10}P$–BTBT and $C_{11}P$–BTBT, Alert level B: PLAT910_ALERT_3_B Missing # of FCF Reflection(s) Below Theta(Min).	These alerts resulted from the large distances of CCD due to the large unit-cell parameters of the crystals.
The structure of C_5P–BTBT, Alert level B: PLAT084_ALERT_3_B High wR2 Value (i.e. > 0.25).	The high wR2 indice for C_5P–BTBT was due to its poor crystallinity.

Supplementary Table 5: *d*-values calculated from GIXD and total facet area computed by morphology modules.

Molecule	% Total facet area of the stable surfaces computed by morphology modules	*d*-values calculated from GIXD and its corresponding faces	
		Vertical	Horizontal
C_3P–BTBT	(001) 60%; (10 $\bar{1}$) 16%	30.8 Å (001)	17 Å -----
C_4P–BTBT	(001) 65.8%; (100) 19.8%	22.7 Å (001)	-----
C_5P–BTBT	(010) 48.1%; (100) 38.2%	23.6 Å (020)	14.7 Å-----
C_6P–BTBT	(010) 32%; (001) 24%	19.1 Å (001)	12.9 Å-----
C_7P–BTBT	(010) 59%; (100) 24%	26.2 Å (020)	20.8 Å-----
C_8P–BTBT	(010) 70%; (011) 14%	30.2 Å (020)	11.0 Å (001)
C_9P–BTBT	(100) 68%; (110) 20%	31.4 Å (100)	15.1 Å (010)
C_{10}P–BTBT	(001) 65%; (102) 22%	33.2 Å (002)	14.9 Å (100)
C_{11}P–BTBT	(100) 64%; (110) 25%	33.4 Å (100)	14.9 Å (010)

Supplementary Notes

Supplementary Note 1 │ Synthetic Procedures and Characterisations

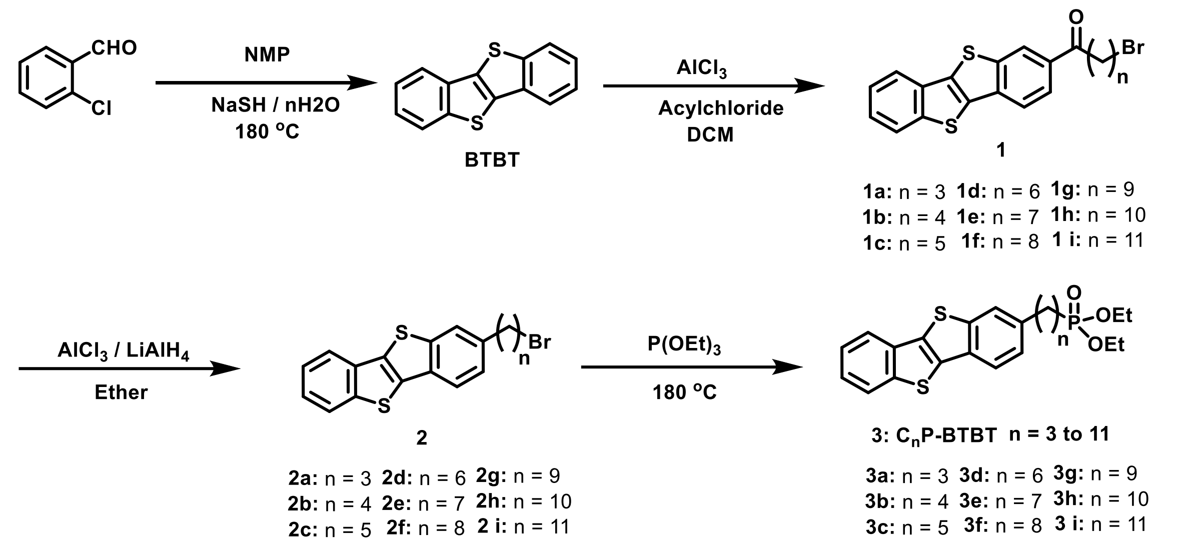

Supplementary Figure 49 │ Synthetic route of C_nP–BTBT (n = 3 to 11).

Benzothieno[3,2–b][1]benzothiophene (BTBT) was synthesised according to the literature[2].

Compound 1. A stirred solution of BTBT (8.197 mmol) in dichloromethane (200 ml) was cooled to –20 °C and aluminium chloride (29.474 mmol) was added in one portion. After cooling to –70 °C, acylchloride (32.782 mmol) was added dropwise within 15 min and the mixture was stirred for 4 h at the given temperature, decomposed with water (100 ml) and diluted with dichloromethane (150 ml) to dissolve all the solid. The organic layer was separated and washed with water (2 × 100 ml), brine (200 ml), and dried with anhydrous magnesium sulfate. Separation was achieved by fractional crystallisation from toluene and purification by column chromatography on silica gel (toluene).

Compound 2. To a stirred suspension of $AlCl_3$ (7.200 mmol) in 10 mL of anhydrous Et_2O, $LiAlH_4$ (7.211 mmol) was added portionwise. To the resulting milky reaction mixture, a solution of 1 (2.404 mmol) in 10 mL of anhydrous DCM was slowly added dropwise. The reaction turned orange with a white precipitate and was stirred at room temperature for 1 h. Excess $LiAlH_4$ was quenched by careful addition of crushed ice, then 2.5 M HCl was added

until acidic pH. The reaction was then washed with aqueous NaCl, and the organic phase was collected and dried over $MgSO_4$. Solvent removal gave the crude product as a yellow solid that was purified with column chromatography on silica gel to yield the compound 2 as a white solid.

Compounds C_nP–BTBT. Compound 2 (0.095 mmol) was dissolved in triethyl phosphite (1 mL). The solution was stirred at 180 °C under argon atmosphere overnight. The residual triethyl phosphite was removed under reduced pressure. The desired product was isolated by silica gel column chromatography with petroleum ether and ethyl acetate (petroleum ether/ethyl acetate = 2/3) to give a white solid.

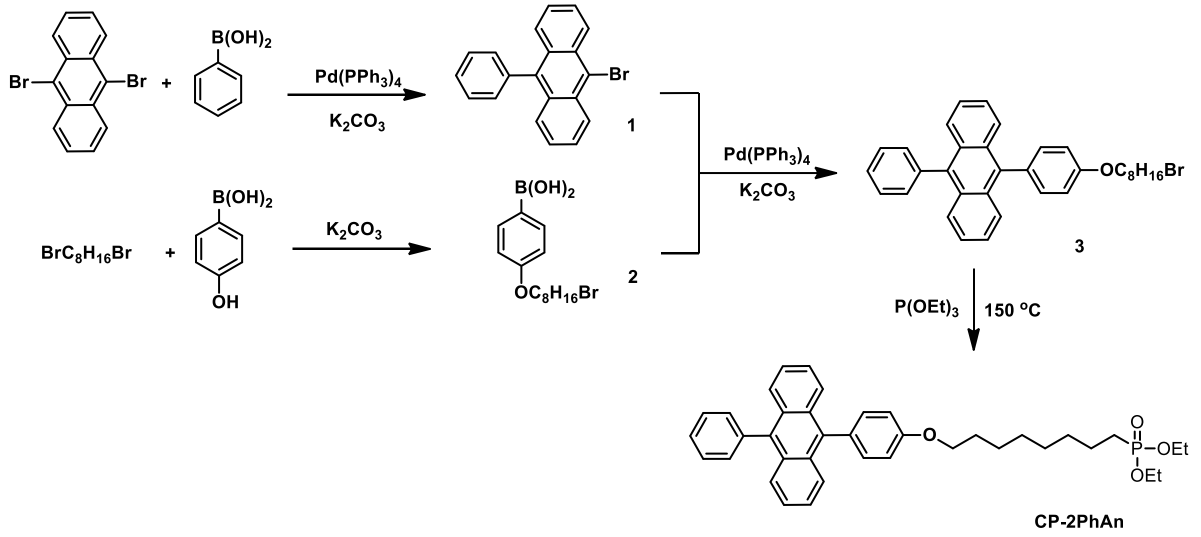

Supplementary Figure 50 │ Synthetic route of CP–2PhAn.

Compound CP–2PhAn. The synthesis routine of CP–2PhAn is shown in Supplementary Figure 50. The target compound was synthesised by a two–step Suzuki reaction. The boronic acid derivative of benzene forms a covalent bond to the 9, 10 sites of anthracene to produce an asymmetric product (Compound 3)[3, 4]. Compound 3 (0.1 mmol) was dissolved in triethyl phosphite (1 mL). The solution was stirred at 150 °C under argon atmosphere overnight. The residual triethyl phosphite was removed under reduced pressure. The desired product was isolated by silica gel column chromatography with petroleum ether and ethyl acetate (petroleum ether/ethyl acetate = 2/3) to give a white solid.

Molecular Characterisations (NMR and MS).

1a (n = 3)

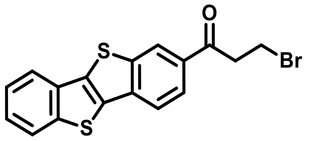

^{1}H NMR ($CDCl_3$, 400 MHz, ppm): δ 8.56 (s, 1H), 8.06 (d, 1H, J=8.3 Hz), 7.96 (m, 3H), 7.49 (m, 2H), 3.81 (t, 2H, J=6.9 Hz), 3.70 (t, 2H, J=6.9 Hz); ^{13}C NMR ($CDCl_3$, 100 MHz, ppm): δ 196.06, 142.92, 142.32, 139.66, 137.47, 136.78, 136.30, 132.69, 126.07, 125.25, 124.69, 124.59, 124.22, 122.21, 121.62, 41.70, 25.88. MALDI-MS: Calcd. for $[M]^{+}$: 373.9435. Found: 373.8900.

1b (n = 4)

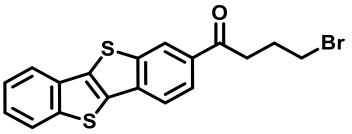

^{1}H NMR ($CDCl_3$, 400 MHz, ppm): δ 8.56 (s, 1H), 8.06 (d, 1H, J=8.3 Hz), 7.93 (m, 3H), 7.47 (m, 2H), 3.60 (t, 2H, J=6.9 Hz), 3.29 (t, 2H, J=6.9 Hz), 2.37 (m, 2H); ^{13}C NMR ($CDCl_3$, 100 MHz, ppm): δ 197.78, 142.86, 142.27, 137.19, 136.50, 133.22, 132.96, 132.72, 125.94, 125.18, 124.59, 124.54, 124.18, 122.12, 121.47, 36.74, 33.68, 26.99. MALDI-MS: Calcd. for $[M]^{+}$: 373.9435. Found: 387.9411.

1c (n = 5)

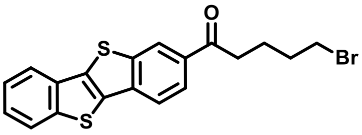

^{1}H NMR ($CDCl_3$, 400 MHz, ppm): δ 8.56 (s, 1H), 8.06 (d, 1H, J=8.3 Hz), 7.95 (m, 3H), 7.49 (m, 2H), 3.49 (t, 2H, J=6.9 Hz), 3.13 (t, 2H, J=6.9 Hz), 1.99 (m, 4H); ^{13}C NMR ($CDCl_3$, 100 MHz, ppm): δ 198.62, 142.84, 142.29, 137.12, 136.44, 133.83, 133.33, 132.74, 125.95, 125.20, 124.66, 124.58, 124.21, 122.14, 121.51, 37.64, 33.43, 32.24, 22.90. MALDI-MS: Calcd. for $[M]^{+}$: 401.9748. Found: 401.8968.

1d (n = 6)

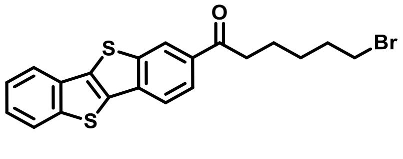

^{1}H NMR ($CDCl_3$, 400 MHz, ppm): *δ* 8.55 (s, 1H), 8.06 (d, 1H, *J*=8.3 Hz), 7.94 (m, 3H), 7.48 (m, 2H), 3.45 (t, 2H, *J*=6.9 Hz), 3.11 (t, 2H, *J*=6.9 Hz), 1.96 (m, 2H), 1.84 (m, 2H), 1.59 (m, 2H); ^{13}C NMR ($CDCl_3$, 100 MHz, ppm): *δ* 198.96, 142.84, 142.28, 137.04, 136.36, 133.49, 132.97, 132.76, 125.89, 125.16, 124.65, 124.52, 124.17, 122.10, 121.43, 38.47, 33.62, 32.66, 27.93, 23.48. ESI-MS: Calcd. for $[M+H]^+$: 416.99770. Found: 416.99715.

1e (n = 7)

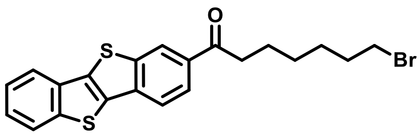

^{1}H NMR ($CDCl_3$, 400 MHz, ppm): *δ* 8.56 (s, 1H), 8.06 (d, 1H, *J*=8.3 Hz), 7.95 (m, 3H), 7.48 (m, 2H), 3.44 (t, 2H, *J*=6.9 Hz), 3.10 (t, 2H, *J*=6.9 Hz), 1.91 (m, 2H), 1.83 (m, 2H), 1.50 (m, 4H); ^{13}C NMR (CDC_{l3}, 100 MHz, ppm): *δ* 199.29, 142.80, 142.24, 137.00, 136.32, 133.51, 132.96, 132.73, 125.88, 125.16, 124.68, 124.55, 124.17, 122.10, 121.44, 38.55, 33.93, 32.58, 28.47, 28.02, 24.17. MALDI-MS: Calcd. for $[M]^+$: 430.0061. Found: 429.9295.

1f (n = 8)

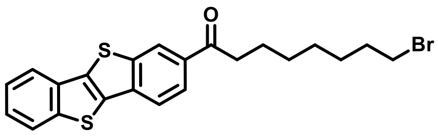

^{1}H NMR ($CDCl_3$, 400 MHz, ppm): *δ* 8.55 (s, 1H), 8.05 (d, 1H, *J*=8.3 Hz), 7.93 (m, 3H), 7.47 (m, 2H), 3.42 (t, 2H, *J*=6.9 Hz), 3.07 (t, 2H, *J*=6.9 Hz), 1.88 (m, 2H), 1.81 (m, 2H), 1.42 (m, 6H); ^{13}C NMR ($CDCl_3$, 100 MHz, ppm): *δ* 199.41, 142.82, 142.26, 136.99, 136.30, 133.58, 133.00, 132.76, 125.88, 125.16, 124.70, 124.55, 124.18, 122.10, 121.43, 38.69, 33.98, 32.76, 29.19, 28.66, 28.04, 24.35. MALDI-MS: Calcd. for $[M]^+$: 444.0217. Found: 443.9394.

1g (n = 9)

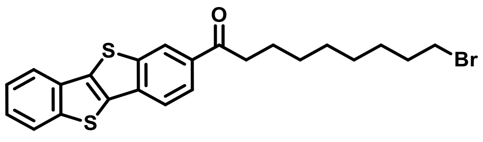

^{1}H NMR ($CDCl_3$, 400 MHz, ppm): *δ* 8.56 (s, 1H), 8.07 (d, 1H, *J*=8.3 Hz), 7.95 (m, 3H), 7.49

(m, 2H), 3.42 (t, 2H, *J*=6.9 Hz), 3.08 (t, 2H, *J*=6.9 Hz), 1.87 (m, 2H), 1.80 (m, 2H), 1.43 (m, 8H); ^{13}C NMR ($CDCl_3$, 100 MHz, ppm): δ 199.53, 142.79, 142.24, 136.96, 136.28, 134.80, 133.57, 132.75, 125.87, 125.16, 124.71, 124.57, 124.17, 122.09, 121.43, 38.75, 34.08, 32.78, 29.31, 29.27, 28.62, 28.12, 24.41. MALDI-MS: Calcd. for $[M]^+$: 458.0374. Found: 457.9386.

1h (n = 10)

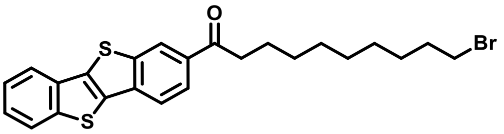

^{1}H NMR ($CDCl_3$, 400 MHz, ppm): δ 8.55 (s, 1H), 8.05 (d, 1H, *J*=8.3 Hz), 7.93 (m, 3H), 7.47 (m, 2H), 3.41 (t, 2H, *J*=6.9 Hz), 3.07 (t, 2H, *J*=6.9 Hz), 1.86 (m, 2H), 1.80 (m, 2H), 1.34 (m, 10H); ^{13}C NMR ($CDCl_3$, 100 MHz, ppm): δ 199.55, 142.82, 142.26, 136.96, 136.28, 133.63, 132.97, 132.77, 125.87, 125.16, 124.72, 124.56, 124.17, 122.09, 121.41, 38.78, 34.06, 32.82, 29.39, 29.35, 29.30, 28.72, 28.16, 24.49. MALDI-MS: Calcd. for $[M]^+$: 472.0530. Found: 472.0058.

1i (n = 11)

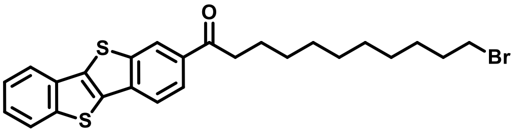

^{1}H NMR ($CDCl_3$, 400 MHz, ppm): δ 8.56 (s, 1H), 8.06 (d, 1H, *J*=8.3 Hz), 7.94 (m, 3H), 7.48 (m, 2H), 3.41 (t, 2H, *J*=6.9 Hz), 3.07 (t, 2H, *J*=6.9 Hz), 1.83 (m, 4H), 1.31 (m, 12H); ^{13}C NMR ($CDCl_3$, 100 MHz, ppm): δ 199.65, 142.81, 142.25, 136.97, 136.29, 133.61, 132.99, 132.77, 125.89, 125.18, 124.74, 124.59, 124.20, 122.12, 121.44, 38.83, 34.17, 32.84, 29.48, 29.42, 28.78, 28.19, 24.52. MALDI-MS: Calcd. for $[M]^+$: 486.0687. Found: 485.9845.

2a (n = 3)

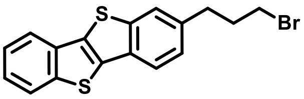

^{1}H NMR ($CDCl_3$, 400 MHz, ppm): δ 7.90 (d, 1H, *J*=7.5 Hz), 7.82 (d, 1H, *J*=7.8 Hz), 7.75 (d, 1H, *J*=8.1 Hz), 7.70 (s, 1H), 7.40 (m, 2H), 7.24 (d, 1H, *J*=8.6 Hz), 3.40 (t, 2H, *J*=6.4 Hz), 2.90 (t, 2H, *J*=7.6 Hz), 2.21 (m, 2H); ^{13}C NMR ($CDCl_3$, 100 MHz, ppm): δ 142.73, 142.15, 137.84, 134.45, 133.18, 132.93, 131.47, 125.88, 124.88, 124.90, 124.04, 122.62, 121.73, 121.50, 35.15, 33.66, 32.23, 30.03. MALDI-MS: Calcd. for $[M]^+$: 359.9642. Found: 359.8948.

2b (n = 4)

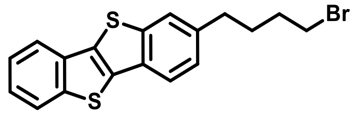

^{1}H NMR ($CDCl_3$, 400 MHz, ppm): δ 7.87 (d, 1H, J=7.8 Hz), 7.83 (d, 1H, J=7.6 Hz), 7.74 (d, 1H, J=8.1 Hz), 7.66 (s, 1H), 7.38 (m, 2H), 7.21 (d, 1H, J=8.1 Hz), 3.40 (t, 2H, J=6.4 Hz), 2.74 (t, 2H, J=7.6 Hz)，1.86 (m, 4H); ^{13}C NMR ($CDCl_3$, 100 MHz, ppm): δ 142.68, 142.14, 139.17, 133.35, 133.23, 132.83, 131.29, 125.79, 124.88, 124.83, 124.04, 123.39, 121.47, 121.44, 35.15, 33.66, 32.23, 30.03. MALDI-MS: Calcd. for $[M]^+$: 373.9799. Found: 373.9390.

2c (n = 5)

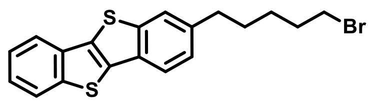

^{1}H NMR ($CDCl_3$, 400 MHz, ppm): δ 7.83 (d, 1H, J=8.0 Hz), 7.78 (d, 1H, J=7.7 Hz), 7.68 (d, 1H, J=8.1 Hz), 7.59 (s, 1H), 7.33 (m, 2H), 7.16 (d, 1H, J=8.1 Hz), 3.32 (t, 2H, J=6.8 Hz), 2.65 (t, 2H, J=7.6 Hz), 1.81 (m, 2H), 1.61 (m, 2H), 1.42 (m, 2H); ^{13}C NMR ($CDCl_3$, 100 MHz, ppm): δ 142.66, 142.15, 139.71, 133.41, 133.27, 132.77, 131.18, 125.84, 124.90, 124.82, 124.05, 123.37, 121.47, 121.36, 35.91, 33.90, 32.74, 30.82, 27.90. MALDI-MS: Calcd. for $[M]^+$: 387.9955. Found: 387.9151.

2d (n = 6)

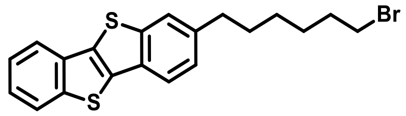

^{1}H NMR ($CDCl_3$, 400 MHz, ppm): δ 7.84 (d, 1H, J=7.9 Hz), 7.79 (d, 1H, J=7.6 Hz), 7.70 (d, 1H, J=8.0 Hz), 7.62 (s, 1 H), 7.35 (m, 2H), 7.17 (d, 1H, J=7.9 Hz), 3.34 (t, 2H, J=6.8 Hz), 2.67 (t, 2H, J=7.6 Hz), 1.79 (m, 2H), 1.63 (m, 2H), 1.35 (m, 4H); ^{13}C NMR ($CDCl_3$, 100 MHz, ppm): δ 142.51, 142.01, 139.84, 133.28, 133.15, 132.58, 131.00, 125.73, 124.73, 124.65, 123.90, 123.23, 121.31, 121.19, 35.84, 33.88, 32.64, 31.30, 28.30, 27.94. ESI-MS: Calcd. for $[M+H]^+$: 403.01843. Found: 403.01924.

2e (n =7)

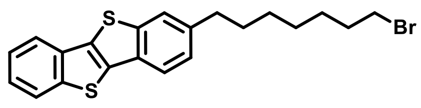

^{1}H NMR ($CDCl_3$, 400 MHz, ppm): δ 7.91 (d, 1H, J=7.9 Hz), 7.86 (d, 1H, J=7.8 Hz), 7.78 (d, 1H, J=8.1 Hz), 7.71 (s, 1H), 7.41 (m, 2H), 7.27 (d, 1H, J=8.2 Hz), 3.40 (t, 2H, J=6.8 Hz), 2.76 (t, 2H, J=7.6 Hz), 1.85 (m, 2H), 1.70 (m, 2H), 1.38 (m, 6H); ^{13}C NMR ($CDCl_3$, 100 MHz, ppm): δ 142.60, 142.09, 140.16, 133.38, 133.26, 132.64, 131.08, 125.90, 124.84, 124.76, 124.02, 123.38, 121.44, 121.32, 36.08, 34.05, 32.79, 31.58, 29.08, 28.66, 28.11. MALDI-MS: Calcd. for $[M]^+$: 416.0268. Found: 415.9489.

2f (n = 8)

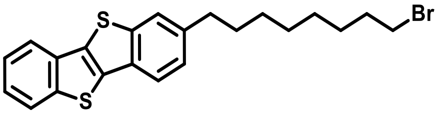

^{1}H NMR ($CDCl_3$, 400 MHz, ppm): δ 7.88 (d, 1H, J=7.8 Hz), 7.83 (d, 1H, J=7.3 Hz), 7.75 (d, 1H, J=8.1 Hz), 7.68 (s, 1H), 7.39 (m, 2 H), 7.24 (d, 1H, J=8.1 Hz), 3.38 (t, 2H, J=6.8 Hz), 2.72 (t, 2H, J=7.6 Hz), 1.82 (m, 2H), 1.67 (m, 2H), 1.32 (m, 8H); ^{13}C NMR ($CDCl_3$, 100 MHz, ppm): δ 142.61, 142.10, 140.25, 133.41, 133.28, 132.64, 131.07, 125.91, 124.84, 124.75, 124.02, 123.38, 121.43, 121.30, 36.12, 34.09, 32.84, 31.65, 29.35, 29.20, 28.73, 28.19. MALDI-MS: Calcd. for $[M]^+$: 430.0425. Found: 430.0033.

2g (n = 9)

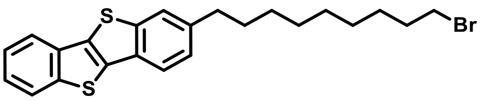

^{1}H NMR ($CDCl_3$, 400 MHz, ppm): δ 7.91 (d, 1H, J=8.0 Hz), 7.86 (d, 1H, J=7.8 Hz), 7.79 (d, 1H, J=8.1 Hz), 7.71 (s, 1H), 7.41 (m, 2H), 7.28 (d, 1H, J=8.1 Hz), 3.40 (t, 2H, J=6.8 Hz), 2.75 (t, 2H, J=7.6 Hz), 1.84 (m, 2H), 1.69 (m, 2H), 1.33 (m, 10H); ^{13}C NMR ($CDCl_3$, 100 MHz, ppm): δ 142.59, 142.08, 140.32, 133.39, 133.27, 132.61, 131.04, 125.92, 124.84, 124.74, 124.02, 123.38, 121.43, 121.30, 36.13, 34.10, 32.83, 31.68, 29.41, 29.37, 29.24, 28.76, 28.17. MALDI-MS: Calcd. for $[M]^+$: 444.0581. Found: 443.9793.

2h (n = 10)

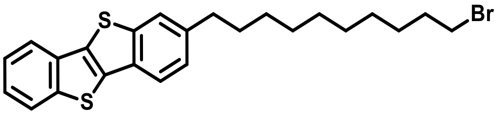

^{1}H NMR ($CDCl_3$, 400 MHz, ppm): δ 7.90 (d, 1H, J=7.9 Hz), 7.86 (d, 1H, J=7.8 Hz), 7.78 (d, 1H, J=8.1 Hz), 7.71 (s, 1H), 7.41 (m, 2H), 7.27 (d, 1H, J=8.1 Hz), 3.39 (t, 2H, J=6.8 Hz), 2.75 (t, 2H, J=7.6 Hz), 1.84 (m, 2H), 1.69 (m, 2H), 1.28 (m, 12H); ^{13}C NMR ($CDCl_3$, 100 MHz,

ppm): δ 142.59, 142.09, 140.35, 133.40, 133.27, 132.61, 131.04, 125.92, 124.83, 124.74, 124.02, 123.38, 121.43, 121.29, 36.14, 34.11, 32.85, 31.69, 29.48, 29.47, 29.43, 29.27, 28.77, 28.19. MALDI-MS: Calcd. for $[M]^+$: 458.0738. Found: 458.0404.

2i (n = 11)

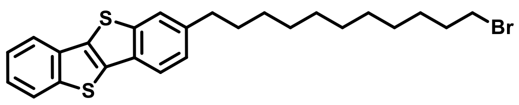

^{1}H NMR ($CDCl_3$, 400 MHz, ppm): δ 7.90 (d, 1H, *J*=7.9 Hz), 7.85 (d, 1H, *J*=7.8 Hz), 7.78 (d, 1H, *J*=8.1 Hz), 7.71 (s, 1 H), 7.41 (m, 2 H), 7.27 (d, 1H, *J*=8.1 Hz), 3.39 (t, 2H, *J*=6.8 Hz), 2.75 (t, 2H, *J*=7.6 Hz), 1.83 (m, 2H), 1.69 (m, 2H), 1.27 (m, 14H); ^{13}C NMR ($CDCl_3$, 100 MHz, ppm): δ 142.59, 142.09, 140.37, 133.40, 133.27, 132.60, 131.03, 125.93, 124.84, 124.74, 124.02, 123.39, 121.43, 121.28, 36.15, 34.14, 32.86, 31.71, 29.54, 29.52, 29.45, 29.30, 28.79, 28.20. MALDI-MS: Calcd. for $[M]^+$: 472.0894. Found: 472.0002.

3a (n = 3)

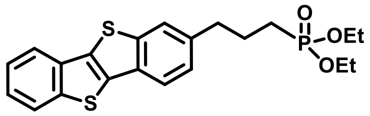

^{1}H NMR ($CDCl_3$, 400 MHz, ppm): δ 7.91 (d, 1H, *J*=8.0 Hz), 7.86 (d, 1H, *J*=7.8 Hz), 7.79 (d, 1H, *J*=8.1 Hz), 7.72 (s, 1H), 7.42 (m, 2H), 7.27 (d, 1H, *J*=7.7 Hz), 4.08 (m, 4H), 2.86 (t, 2H, *J*=7.4 Hz), 2.02 (m, 2H), 1.78 (m, 2H), 1.31 (m, 6H); ^{13}C NMR ($CDCl_3$, 100 MHz, ppm): δ 142.68, 142.13, 138.41, 133.29, 133.20, 132.91, 131.42, 125.85, 124.87, 124.85, 124.02, 123.58, 121.49, 121.48, 61.54, 61.48, 36.63, 36.47, 25.82, 24.41, 24.40, 24.35, 16.54, 16.48. ESI-MS: Calcd. for $[M+H]^+$: 419.069900. Found: 419.089829. Elemental Anal. Calcd. for $C_{21}H_{23}O_3PS_2$: C, 60.27; H, 5.54. Found: C, 60.21; H, 5.56.

3b (n = 4)

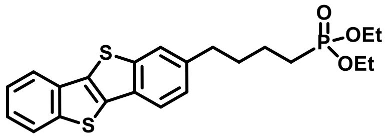

^{1}H NMR ($CDCl_3$, 400 MHz, ppm): δ 7.91 (d, 1H, *J*=8.0 Hz), 7.86 (d, 1H, *J*=7.8 Hz), 7.79 (d, 1H, *J*=8.1 Hz), 7.71 (s, 1H), 7.42 (m, 2H), 7.27 (d, 1H, *J*=8.0 Hz), 4.08 (m, 4H), 2.79 (t, 2H, *J*=7.4 Hz), 1.75 (m, 6H), 1.31 (m, 6H); ^{13}C NMR ($CDCl_3$, 100 MHz, ppm): δ 142.65, 142.11, 138.36, 133.33, 133.23, 132.76, 131.22, 125.83, 124.86, 124.80, 124.02, 123.41, 121.46,

121.41, 61.51, 61.44, 35.60, 32.57, 32.41, 26.31, 24.91, 22.16, 22.11, 16.53, 16.47. ESI-MS: Calcd. for $[M+H]^+$: 433.105550. Found: 433.106656. Elemental Anal. Calcd. for $C_{22}H_{25}O_3PS_2$: C, 61.09; H, 5.83. Found: C, 60.96; H, 5.89.

3c (n = 5)

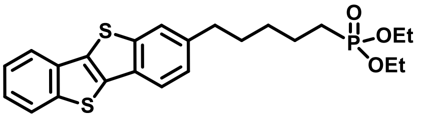

^{1}H NMR ($CDCl_3$, 400 MHz, ppm): δ 7.80 (d, 1H, *J*=7.9 Hz), 7.75 (d, 1H, *J*=7.8 Hz), 7.65 (d, 1H, *J*=8.1 Hz), 7.57 (s, 1H), 7.30 (m, 2H), 7.14 (d, 1H, *J*=8.1 Hz), 4.05 (m, 4H), 2.64 (t, 2H, *J*=7.5 Hz), 1.63 (m, 6H), 1.36 (m, 2H), 1.27 (m, 6H); ^{13}C NMR ($CDCl_3$, 100 MHz, ppm): δ 142.55, 142.04, 139.72, 133.30, 133.16, 132.63, 131.04, 125.75, 124.78, 124.70, 123.92, 123.25, 121.32, 121.22, 61.41, 61.35, 35.74, 31.05, 30.21, 30.04, 26.28, 24.89, 22.38. 22.33, 16.55, 16.49. ESI-MS: Calcd. for $[M+H]^+$: 447.121200. Found: 447.122221. Elemental Anal. Calcd. for $C_{23}H_{27}O_3PS_2$: C, 61.86; H, 6.09. Found: C, 61.86; H, 5.99.

3d (n = 6)

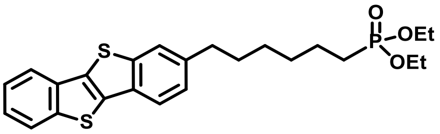

^{1}H NMR ($CDCl_3$, 400 MHz, ppm): δ 7.91 (d, 1H, *J*=8.0 Hz), 7.86 (d, 1H, *J*=7.8 Hz), 7.79 (d, 1H, *J*=8.1 Hz), 7.71 (s, 1H), 7.42 (m, 2H), 7.27 (d, 1H, *J*=7.9 Hz), 4.09 (m, 4H), 2.76 (t, 2H, *J*=7.5 Hz), 1.70 (m, 4H), 1.62 (m, 2H), 1.39 (m, 4H), 1.31 (m, 6H); ^{13}C NMR ($CDCl_3$, 100 MHz, ppm): δ 142.59, 142.08, 140.06, 133.36, 133.24, 132.65, 131.08, 125.89, 124.84, 124.76, 124.02, 123.38, 121.44, 121.32, 61.44, 61.37, 36.04, 31.42, 30.58, 30.41, 28.76, 26.39, 24.99. 22.42, 22.37, 16.55, 16.49. ESI-MS: Calcd. for $[M+H]^+$: 461.136850. Found: 461.137170. Elemental Anal. Calcd. for $C_{24}H_{29}O_3PS_2$: C, 62.58; H, 6.35. Found: C, 62.48; H, 6.40.

3e (n = 7)

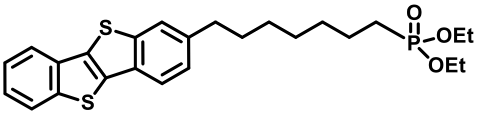

^{1}H NMR ($CDCl_3$, 400 MHz, ppm): δ 7.74 (d, 1H, *J*=7.9 Hz), 7.67 (d, 1H, *J*=7.7 Hz), 7.56 (d, 1H, *J*=8.0 Hz), 7.47 (s, 1H), 7.26 (m, 2H), 7.05 (d, 1H, *J*=8.1 Hz), 4.01 (m, 4H), 2.53 (t, 2H, *J*=7.5 Hz), 1.51 (m, 6H), 1.24 (m, 12H); ^{13}C NMR ($CDCl_3$, 100 MHz, ppm): δ 142.43, 141.94,

139.93, 133.23, 133.08, 132.48, 130.85, 125.66, 124.66, 124.55, 123.79, 123.08, 121.17, 121.02, 61.27, 61.20, 35.89, 31.40, 30.51, 30.34, 28.97, 28.93, 26.25, 24.85, 22.37, 22.32, 16.50, 16.45. ESI-MS: Calcd. for $[M+H]^+$: 475.152500. Found: 475.152438. Elemental Anal. Calcd. for $C_{25}H_{31}O_3PS_2$: C, 63.27; H, 6.58. Found: C, 63.39; H, 6.68.

3f (n = 8)

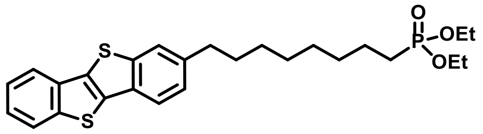

^{1}H NMR ($CDCl_3$, 400 MHz, ppm): δ 7.90 (d, 1H, *J*=8.0 Hz), 7.86 (d, 1H, *J*=7.8 Hz), 7.78 (d, 1H, *J*=8.1 Hz), 7.71 (s, 1H), 7.42 (m, 2H), 7.27 (d, 1H, *J*=9.1 Hz), 4.08 (m, 4H), 2.75 (t, 2H, *J*=7.5 Hz), 1.69 (m, 4H), 1.60 (m, 2H), 1.31 (m, 14H); ^{13}C NMR ($CDCl_3$, 100 MHz, ppm): δ 142.59, 142.09, 140.28, 133.39, 133.27, 132.62, 131.05, 125.91, 124.83, 124.74, 124.01, 123.38, 121.42, 121.29, 61.42, 61.35, 36.11, 31.65, 30.70, 30.53, 29.27, 29.22, 29.06, 26.42, 25.03, 22.45, 22.40, 16.54, 16.48. ESI-MS: Calcd. for $[M+H]^+$: 489.168150. Found: 489.169354. Elemental Anal. Calcd. for $C_{26}H_{33}O_3PS_2$: C, 63.91; H, 6.81. Found: C, 63.86; H, 6.90.

3g (n = 9)

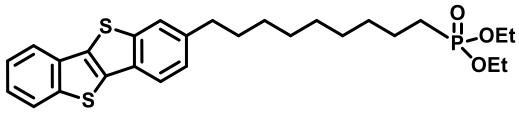

^{1}H NMR ($CDCl_3$, 400 MHz, ppm): δ 7.90 (d, 1H, *J*=7.9 Hz), 7.86 (d, 1H, *J*=7.6 Hz), 7.78 (d, 1H, *J*=8.1 Hz), 7.71 (s, 1H), 7.41 (m, 2H), 7.27 (d, 1H, *J*=8.2 Hz), 4.07 (m, 4H), 2.75 (t, 2H, *J*=7.6 Hz), 1.71 (m, 4H), 1.59 (m, 2H), 1.31 (m, 16H); ^{13}C NMR ($CDCl_3$, 100 MHz, ppm): δ 142.59, 142.08, 140.33, 133.39, 133.27, 132.60, 131.04, 125.91, 124.83, 124.73, 124.01, 123.37, 121.42, 121.29, 61.41, 61.35, 36.13, 31.69, 30.71, 30.54, 29.46, 29.33, 29.27, 29.11, 26.41, 25.02, 22.45, 22.40, 16.54, 16.48. ESI-MS: Calcd. for $[M+H]^+$: 503.183800. Found: 503.184064. Elemental Anal. Calcd. for $C_{27}H_{35}O_3PS_2$: C, 64.51; H, 7.02. Found: C, 64.53; H, 7.04.

3h (n = 10)

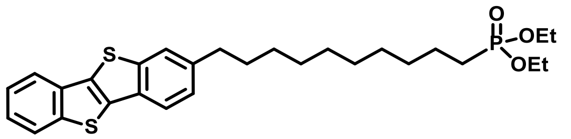

^{1}H NMR ($CDCl_3$, 400 MHz, ppm): δ 7.91 (d, 1H, J=7.9 Hz), 7.86 (d, 1H, J=7.8 Hz), 7.79 (d, 1H, J=8.1 Hz), 7.71 (s, 1H), 7.42 (m, 2H), 7.28 (d, 1H, J=8.2 Hz), 4.08 (m, 4H), 2.76 (t, 2H, J=7.6 Hz), 1.71 (m, 4H), 1.59 (m, 2H), 1.32 (m, 18H); ^{13}C NMR ($CDCl_3$, 100 MHz, ppm): δ 142.58, 142.08, 140.37, 133.39, 133.27, 132.60, 131.03, 125.92, 124.82, 124.73, 124.01, 123.37, 121.42, 121.28, 61.40, 61.34, 36.14, 31.70, 30.71, 30.54, 29.51, 29.49, 29.38, 29.29, 29.10, 26.42, 25.03, 22.40, 16.53, 16.47. ESI-MS: Calcd. for $[M+H]^+$: 517.199450. Found: 517.198916. Elemental Anal. Calcd. for $C_{28}H_{37}O_3PS_2$: C, 65.09; H, 7.22. Found: C, 65.28; H, 7.33.

3i (n = 11)

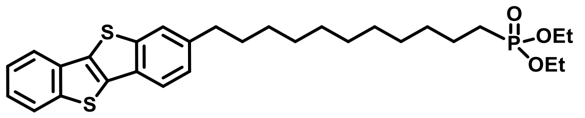

^{1}H NMR ($CDCl_3$, 400 MHz, ppm): δ 7.85 (d, 1H, J=7.9 Hz), 7.80 (d, 1H, J=7.8 Hz), 7.72 (d, 1H, J=8.1 Hz), 7.65 (s, 1H), 7.36 (m, 2H), 7.21 (d, 1H, J=8.1 Hz), 4.07 (m, 4H), 2.70 (t, 2H, J=7.6 Hz), 1.65 (m, 6H), 1.30 (m, 20H); ^{13}C NMR ($CDCl_3$, 100 MHz, ppm): δ 142.59, 142.08, 140.38, 133.39, 133.27, 132.60, 131.03, 125.92, 124.82, 124.72, 124.01, 123.37, 121.42, 121.27, 61.41, 61.34, 36.15, 31.71, 30.73, 30.56, 29.59, 29.56, 29.53, 29.39, 29.31, 29.12, 26.42, 25.02, 22.45, 22.40, 16.54, 16.48. ESI-MS: Calcd. for $[M+H]^+$: 531.215100. Found: 531.215006. Elemental Anal. Calcd. for $C_{29}H_{39}O_3PS_2$: C, 65.63; H, 7.41. Found: C, 65.55; H, 7.38.

CP–2PhAn

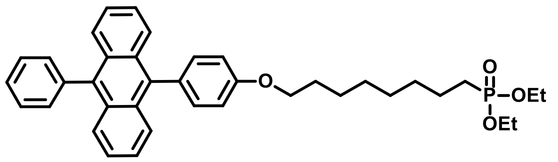

^{1}H NMR ($CDCl_3$, 400 MHz, ppm): δ 7.64 (q, 2H, J=3.4 Hz), 7.56 (q, 2H, J=3.4 Hz), 7.44 (d, 2H, J=7.3 Hz), 7.40 (d, 1H, J=7.0 Hz), 7.34 (d, 2H, J=6.76 Hz), 7.24 (d, 2H, J=8.44 Hz), 7.18 (m, 4H), 6.98 (d, 2H, J=8.4 Hz), 3.97 (m, 6H), 1.21(m, 22H); ^{13}C NMR ($CDCl_3$, 100 MHz, ppm): δ 157.52, 138.06, 135.94, 135.79, 131.27, 130.24, 129.78, 129.14, 128.84, 127.33, 126.36, 126.00, 125.85, 123.90, 123.81, 113.32, 66.94, 60.40, 60.34, 29.57, 29.40, 28.30, 28.12, 28.02, 25.32, 25.06, 23.92, 21.38, 21.33, 15.48, 15.42. ESI-MS: Calcd. for $[M+H]^+$: 595.297173. Found 595.298592.

Supplementary Note 2 │ AFM Data Processing and Code

Separating touching objects in an image is one of the more difficult image processing operations. The watershed transform is often applied to solve this problem. The watershed transform finds "catchment basins" and "watershed ridge lines" in an image by treating it as a surface where light pixels are high and dark pixels are low. Segmentation by using the watershed transform works better if you can identify, or "mark," foreground objects and background locations. Marker-controlled watershed segmentation follows this basic procedure:

Step 1: Read in the color image and convert it to a grayscale binary image.

Step 2: Use the gradient magnitude as the segmentation function.

Step 3: Mark the foreground objects.

A variety of procedures could be applied here to find the foreground markers, which must be connected blobs of pixels inside each of the foreground objects. In this example, you can use morphological techniques called "opening-by-reconstruction" and "closing-by-reconstruction" to "clean" up the image. These operations will create flat maxima inside each object that can be located by using imregionalmax.

Step 4: Compute background markers.

Step 5: Compute the watershed transform of the segmentation function.

The function imimposemin can be used to modify an image so that it has regional minima only in certain desired locations. We can use imimposemin to modify the gradient magnitude image so that its only regional minima occur at foreground and background marker pixels.

Step 6: Visualise the results.

Marker-based watershed segmentation Matlab code:

```
clc;clear;close all;
rgb=imread('S-4-2.jpg');
  if ndims(rgb) == 3
        I = rgb2gray(rgb);
else
        I = rgb;
end

hy = fspecial('sobel');
hx = hy';
Iy = imfilter(double(I), hy, 'replicate');
Ix = imfilter(double(I), hx, 'replicate');
gradmag = sqrt(Ix.^2 + Iy.^2);

%Step3:
se=strel('disk',3);
Io=imopen(I,se);

Ie = imerode(I, se);
Iobr = imreconstruct(Ie, I);
  Ioc = imclose(Io, se);
Ic = imclose(I, se);
  Iobrd = imdilate(Iobr, se);
Iobrcbr = imreconstruct(imcomplement(Iobrd), imcomplement(Iobr));
Iobrcbr = imcomplement(Iobrcbr);
  fgm = imregionalmax(Iobrcbr);
It1 = rgb(:, :, 1);
It2 = rgb(:, :, 2);
It3 = rgb(:, :, 3);
It1(fgm) = 255; It2(fgm) = 0; It3(fgm) = 0;
I2 = cat(3, It1, It2, It3);
  se2 = strel(ones(3,3));
fgm2 = imclose(fgm, se2);
fgm3 = imerode(fgm2, se2);
  fgm4 = bwareaopen(fgm3, 20);
It1 = rgb(:, :, 1);
It2 = rgb(:, :, 2);
It3 = rgb(:, :, 3);
It1(fgm4) = 255; It2(fgm4) = 0; It3(fgm4) = 0;
I3 = cat(3, It1, It2, It3);
  %Step4,
bw = im2bw(Iobrcbr, graythresh(Iobrcbr));
D = bwdist(bw);
DL = watershed(D);
bgm = DL == 0;
%Step5,
gradmag2 = imimposemin(gradmag, bgm | fgm4);
L = watershed(gradmag2);
  %Step6,
It1 = rgb(:, :, 1);
It2 = rgb(:, :, 2);
It3 = rgb(:, :, 3);
fgm5 = imdilate(L == 0, ones(3, 3)) | bgm | fgm4;
It1(fgm5) = 255; It2(fgm5) = 0; It3(fgm5) = 0;
I4 = cat(3, It1, It2, It3);

Lrgb = label2rgb(L, 'jet', 'w', 'shuffle');

sats=regionprops(bw,'Area','Centroid','Image','Filledimage','FilledArea');
centroids = cat(1, sats.Centroid);
areatot = cat(1, sats.Area);
  bin = 100;
[counts,areanum] = hist(areatot,bin);
output(:,1) = areanum;
output(:,2) = counts;
  save histfit.txt -ascii output
  figure('units', 'normalized', 'position', [0 0 1 1]);
  subplot(2, 2, 1); hist(areatot,bin);hold on;
  subplot(2, 2, 2); imshow(bw, []); title('Thresholding');hold on;
plot(centroids(:,1),centroids(:,2), 'b*')
  subplot(2, 2, 3); imshow(I4, []); title('Edge superimpose to Original');
subplot(2, 2, 4); imshow(rgb, []); hold on;
himage = imshow(Lrgb);
set(himage, 'AlphaData', 0.3);
title('Watershed superimpose to Original');hold on;

plot(centroids(:,1),centroids(:,2), 'b*')
```

Supplementary Note 3 | Kinetic Monte Carlo simulation

(I) Methods

We used kinetic Monte Carlo (KMC) methods to simulate the crystal growth. A 200 × 200 square lattice with periodic boundary condition based on the terrace–step–kink (TSK) model is used. Initially, N_a molecules are randomly spread on the lattice. The coverage is defined as:

$$\eta_c = \frac{N_a}{N^2} \times 100\% \tag{1}$$

where N is the lattice size (here N = 200).

According to the Arrhenius equation, the rate constant for a molecule hopping from site *A* to *B* is given by

$$k = f_0 \exp\left(-\frac{U_{AB}}{k_B T}\right) \tag{2}$$

where f_0 is the pre-exponential factor and U_{AB} is the activation barrier from site *A* to *B*.

In the simulation, a molecule can hop into both four nearest-neighbor sites and four next-nearest-neighbor sites. We settled different activation barriers including the bond barrier, the terrace barrier and the detachment barrier based on different local environments and hopping mechanisms of the molecules. In our model, the activation barrier has the following forms in different situations:

$$U_{AB} = U_{terrace} + n * U_{detachment} \tag{3}$$

when molecules detach from the island edges where *n* denotes the number of lateral bonds at site *A*, and

$$U_{AB} = U_{terrace} + n * U_{bond} \tag{4}$$

when molecules diffuse along the island edges where *n* denotes the number of lateral bonds at site *A*, and

$$U_{AB} = U_{terrace} \tag{5}$$

when molecules diffuse on the terrace plane. Here, an island is referred to any connected cluster whose size is over a given value.

In our implementation, we accelerate the algorithm by using the time interval of per terrace diffusion, the fastest kinetic process in our model, as the unit time. Therefore, Supplementary Equation 2 is further modified as

$$k = f_0 \exp\left(-\frac{U_{AB} - U_{terrace}}{k_B T}\right) \tag{6}$$

(II) Terrace and detachment barrier effects.

Here, we first introduce two main quantitative criteria, island and overlap percentage, to characterise the overall morphology and mobility of islands. Island percentage is defined as

$$\eta_i \equiv \frac{N_s}{N_a} \tag{7}$$

where N_s is the number of molecules which belong to an island (here we only count islands whose size is over a given critical size) and N_a is the total number of molecules. Overlap percentage is defined as

$$\eta_o = \frac{N_o}{N_a} \times 100\% \tag{8}$$

where N_o is the overlapped area between half time- and final time-morphology of islands. Besides, we can introduce another two parameters, total number of islands (T) and average island size ($\bar{S}$)

$$T = \Sigma_i 1, \text{where } S_i > \text{critical size} \tag{9}$$

$$\bar{S} = \frac{S_{total}}{T} \tag{10}$$

Because any overall kinetics of a system is governed by its own set of physical parameters (like barriers, temperatures and etc.), we can map an experimental system to a point in a high dimensional parameter space. Therefore, in order to compare different systems, we can simply tune the parameters and verify the effects. Specifically, in our case, molecules with different number of carbon atoms in the middle chain are represented by different sets of barrier parameters so that we can compare them by seeing how the overall morphology and evolution of islands are influenced by these parameters. Here, we focus on the effects of two barriers, the terrace and detachment barriers.

A. The terrace barrier

We fix the detachment barrier to a given value and change the terrace barrier to obtain its effects on the crystal growth and overall morphology of the islands. In general, the island percentage decreases as the terrace barrier increases because the higher terrace barrier means lower diffusion rates, which hinder the formation of islands. This is consistent with the results obtained from the overlap percentage curve, which has a monotonous increasing tendency (Supplementary Figure 32).

B. The detachment barrier

We fix the terrace barrier to a given value and change the detachment barrier to see its effects on the growth and morphologies of islands. From the simulation results, we find that it has a volcano-like influence that the island percentage is lower either in the case of low or high detachment barrier than that with a mediate detachment barrier (Supplementary Figure 33). However, it is worth noting here that the two lower island percentage sides are under totally different mechanisms. It can be simply understood through a two-extreme consideration. When the detachment barrier is too high, molecules are nearly impossible to detach from the island's edges so that the evolution is limited. On the other hand, when the detachment barrier is too low, molecules can easily detach, resulting in the disassembly of the islands.

C. The total effect

It is shown in Supplementary Figure 34 that terrace barrier has a monotonous while detachment barrier has a volcano-like influence on the overall morphology and evolution of islands in the case of using island percentage as the characterising criterion. Therefore, the parameters in together, rather than alone, determine the growth process in a system.

(III) Binding energy calculation

The first–principles calculations were performed by using the Vienna Ab Initio Simulation Package (VASP)[5], which is based on the density functional theory and plane wave basis sets with the projector–augmented wave (PAW) method[6, 7]. The exchange and correlation functional was treated by using the Perdew–Burke–Ernzerhof (PBE)[8] parameterisation of generalised gradient approximation (GGA), including the van der Waals corrections as parameterised in the semiempirical DFT–D3 method[9], for total energy calculations. ISIF = 3 was used for structural relaxations of organic molecular crystals, and vacuum regions of more than 15 Å were used in each axis for a single organic molecule calculation. The outmost *s* and *p* electrons for P, S, O, and C were treated as valence states in the PAW potentials, and ultrasoft pseudopotentials were used for H. The energy cut–off of the plane wave basis was set as 700 eV. Electronic minimisation was performed with a tolerance of 10^{-4} eV, and ionic relaxation was performed with a force tolerance of 0.01 eV/Å on each ion. All these parameters were carefully tested to ensure the convergence and accuracy. The binding energy $\Delta E = E(\text{crystal})/n - E(\text{molecule})$, where E(crystal) and E(molecule) are the total energy of the organic molecular crystals and single organic molecules, respectively, and n is the number of organic molecules in the crystals.

Supplementary References

1. Sheldrick, G. M. A short history of SHELX. *Acta Cryst. A* **64**, 112–122 (2008).
2. Saito M. *et al.* One–step synthesis of [1]benzothieno[3,2–b][1]benzothiophene from *o*–chlorobenzaldehyde. *Tetrahedron Lett.* **52**, 285–288 (2011).
3. Chung, S.–J., Jin, J.–I. & Kim, K.–K. Novel PPV derivatives emitting light over a broad wavelength range. *Adv. Mater.* **9**, 551–554 (1997).
4. Chung, S.–J., Kim, K.–K. & Jin, J.–I. Fluorescing wholly aromatic polyesters containing diphenylanthracene fluorophores. *Polymer* **40**, 1943–1953 (1999).
5. Kresse, G. & Furthmüller, J. Efficient iterative schemes for *ab initio* total–energy calculations using a plane–wave basis set. *Phys. Rev. B* **54**, 11169–11186 (1996).
6. Blöchl, P. E. Projector augmented–wave method. *Phys. Rev. B* **50**, 17953–17979 (1994).
7. Kresse, G. & Joubert, D. From ultrasoft pseudopotentials to the projector augmented–wave method. *Phys. Rev. B* **59**, 1758–1775 (1999).
8. Perdew, J. P., Burke, K. & Ernzerhof, M. Generalized gradient approximation made simple. *Phys. Rev. Lett.* **77**, 3865–3868 (1996).
9. Grimme, S., Antony, J., Ehrlich, S. & Krieg, H. A consistent and accurate *ab initio* parametrization of density functional dispersion correction (DFT-D) for the 94 elements H-Pu. *J. Chem. Phys.* **132**, 154104 (2010).
