# Supplementary material for: Multistep nucleation and growth mechanisms of organic crystals from amorphous solid states
Source: Nat Commun. 2019 Aug 27;10:3872. doi: 10.1038/s41467-019-11887-2 (PMC6711996; doi:10.1038/s41467-019-11887-2)
Supplement: Supplementary file 3 — Description of Additional Supplementary Files [file 41467_2019_11887_MOESM3_ESM.pdf]

Description of Additional Supplementary Files

File Name: Supplementary Movie 1
Description: The prenucleation process The movie shows a top view of the prenucleation process when a liquid droplet is cast on a flat SiO2 surface. The semiconducting material used is 0.5 mg/mL C7P–BTBT to prevent the films from coalescence. Driven by the interfacial energy and hydrostatic pressure, PNCs are flattened into a pancake–like structure, spreading to the SiO2 surface, and increasing in area. We can clearly observe the coalescence and fusion of two adjacent PNCs, indicating a non–classical nucleation mechanism.

File Name: Supplementary Movie 2
Description: Spinodal decomposition The movie shows a top view of the spinodal decomposition process, explaining how small density fluctuations can cause a homogeneous film and demix into metastable high–entropy nucleus–poor and low–enthalpy nucleus–rich films. The semiconducting material used is 1.0 mg/mL C7P–BTBT.

File Name: Supplementary Movie 3
Description: Film formation Real–time imaging of the system evolution at a 1:10000 fluorescent molecular probe content by using the conventional mode of a Nikon Stochastic Optical Reconstruction Microscopy (N– STORM). The semiconducting material used is 1.0 mg/mL C7P–BTBT

File Name: Supplementary Movie 4
Description: Mass transport The movie shows a top view of the Ostwald ripening process, in which the “self–selection” process of the fast–growing nucleus–rich domains eventually overwhelmed the slow–growing nucleus–poor domains. We can clearly observe the growth of nucleus–poor domains (Part 1) while the nucleus–poor domains (Parts 2–4) disappeared gradually. The semiconducting material used is 1.0 mg/mL C7P–BTBT.

File Name: Supplementary Movie 5
Description: The layer growth mode The movie shows a top view of the self–confined layer growth mode, during which the high energy crystal facet is etched and serves as the molecular source to grow the upper layer. The semiconducting material used is 1.0 mg/mL C7P–BTBT.

File Name: Supplementary Movie 6
Description: Kinetic Monte Carlo simulation Islands morphologies evolution at 106 computation steps.
